# Durum Wheat Roots Adapt to Salinity Remodeling the Cellular Content of Nitrogen Metabolites and Sucrose

**DOI:** 10.3389/fpls.2016.02035

**Published:** 2017-01-09

**Authors:** Maria Grazia Annunziata, Loredana F. Ciarmiello, Pasqualina Woodrow, Eugenia Maximova, Amodio Fuggi, Petronia Carillo

**Affiliations:** ^1^Department of Metabolic Networks, Max Planck Institute of Molecular Plant PhysiologyPotsdam, Germany; ^2^Dipartimento di Scienze e Tecnologie Ambientali, Biologiche e Farmaceutiche, Università degli Studi della Campania “Luigi Vanvitelli”Caserta, Italy

**Keywords:** osmotic adjustment, glycine betaine, asparagine, asparagine synthetase, P5CS, nitrate reductase

## Abstract

Plants are currently experiencing increasing salinity problems due to irrigation with brackish water. Moreover, in fields, roots can grow in soils which show spatial variation in water content and salt concentration, also because of the type of irrigation. Salinity impairs crop growth and productivity by inhibiting many physiological and metabolic processes, in particular nitrate uptake, translocation, and assimilation. Salinity determines an increase of sap osmolality from about 305 mOsmol kg^−1^ in control roots to about 530 mOsmol kg^−1^ in roots under salinity. Root cells adapt to salinity by sequestering sodium in the vacuole, as a cheap osmoticum, and showing a rearrangement of few nitrogen-containing metabolites and sucrose in the cytosol, both for osmotic adjustment and oxidative stress protection, thus providing plant viability even at low nitrate levels. Mainly glycine betaine and sucrose at low nitrate concentration, and glycine betaine, asparagine and proline at high nitrate levels can be assumed responsible for the osmotic adjustment of the cytosol, the assimilation of the excess of ammonium and the scavenging of ROS under salinity. High nitrate plants with half of the root system under salinity accumulate proline and glutamine in both control and salt stressed split roots, revealing that osmotic adjustment is not a regional effect in plants. The expression level and enzymatic activities of asparagine synthetase and Δ1-pyrroline-5-carboxylate synthetase, as well as other enzymatic activities of nitrogen and carbon metabolism, are analyzed.

## Introduction

Salinity affects more than 40% of soils in the Mediterranean basin (Nedjimi, 2014). In this area seawater intrusion into freshwater aquifers and irrigation with brackish water highly contribute to soil salinization (Rana and Katerji, 2000). Indeed, irrigation with salinized water and scarce winter rainfall contribute to further increase the salt stress problems with a significant decrease in crops productivity. In these conditions, crops have to cope with daily exposure to hyperosmotic stress and seasonal effects due to salt accumulation in the roots (Maggio et al., 2011).

Soil salinity inhibits plant growth mainly due to osmotic stress and ion toxicity (Munns and Tester, 2008; Gorham et al., 2010). High salinity decreases the capacity of roots to extract water from soil, and high concentrations of salts within the plant itself can be toxic, resulting in plant nutritional imbalance and oxidative stress (Hasegawa et al., 2000; Munns, 2002; Munns and Tester, 2008). This dual effect reduces plant growth, development, and survival. However, the extent of the damage to crops depends on the concurrent salt toxicity levels and phenological stage sensitivity to salt stress (Lutts et al., 1995; Hasegawa et al., 2000). Seedling stage, for example, is the more vulnerable phase of durum wheat growth under salinity (Carillo et al., 2008). This species, which is mainly cropped in Mediterranean type climate, is more sensitive to salinity than bread wheat (Gorham et al., 1990; James et al., 2006) and yields poorly on saline soil (Munns et al., 2006; Rahnama et al., 2011) partly due to the scarce ability of durum wheat to exclude sodium (Colmer et al., 2006; James et al., 2011). Sodium has, in fact, a damaging effect on cytosol and organelles metabolism because it tends to replace potassium in key enzymatic reactions. For this reason, the potassium to sodium ratio is more critical than the absolute amount of sodium for the cell performance under salinity (Maathuis and Amtmann, 1999; Shabala and Cuin, 2008; Cuin et al., 2009). However, exposure to salinity triggers specific strategies for cell osmotic adjustment and control of ion and water homeostasis to minimize stress damage and to re-establish growth (Hasegawa et al., 2000; Puniran-Hartley et al., 2014; Gao et al., 2016; Woodrow et al., 2016). A ubiquitous mechanism that plants have evolved to adapt to salinity involves sodium sequestration in the vacuole, as a cheap osmoticum, and synthesis and accumulation of compatible compounds, which have a much higher cost in terms of energy needed for their synthesis (50–70 moles ATP for mole), both for osmotic adjustment and oxidative stress protection in the cytosol (Raven, 1985; Cuin et al., 2009; Shabala, 2013). Most of compatible solutes are N-containing metabolites, such as amino acids, amines, and betaines (Mansour, 2000). Therefore, nitrogen availability is of pivotal importance in plants under salinity. This is true not only for growth, but also for the synthesis of these organic solutes involved in osmoprotection (Krishna Rao and Gnanam, 1990; Silveira et al., 2001). Nevertheless, salinity affects root nitrate influx and loading of nitrate into the root xylem (Peuke and Jeschke, 1999), nitrate reductase activity (Abd-El-Baki et al., 2000; Carillo et al., 2005), amino acid metabolism (Silveira et al., 2012), and protein synthesis (Aslam et al., 1996). The imbalance between nitrogen assimilation and protein synthesis under salinity could be responsible for the increase of free amino acids in roots and shoots of plants under salinity (Silveira et al., 2001). In particular, salinity greatly increases the levels of proline and glycine betaine in durum wheat (Munns, 2002; Carillo et al., 2008), as in other Poaceae (Sairam and Tyagi, 2004; Carillo et al., 2005; Ashraf and Foolad, 2007). In many halophytes, leaf concentration of proline, GB or both contributes to the osmotic pressure in the cell as a whole (Flowers et al., 1977). In glycophytes, proline and GB have lower concentrations but, being partitioned exclusively to the cytoplasm, which makes up about <10% of the volume of the cell, they are able to determine significant osmotic pressure and balance the vacuolar osmotic potential (Cuin et al., 2009).

Notwithstanding several studies have already been carried out on durum wheat under salinity, most of them were performed on leaves. Only few data concern the effects of salinity on root metabolic profile, and how metabolite changes are related to the physiology of cells and root tissues (Zubaidi et al., 1999; Maggio et al., 2003; Carillo et al., 2005; Cuin and Shabala, 2007; Cuin et al., 2008). Moreover, plant metabolic response to salt stress can greatly differ depending on environmental factors in the soil. One of these factors is that salinity in the fields is normally distributed in patches (Richards, 1983) and therefore heterogeneous (Sonneveld and de Kreij, 1999; Kong et al., 2012). Experiments carried out in hydroponics, a homogenous environment, and in soils have given contrasting results. It has been argued, thus, that it is more realistic to study the effects of salinity in heterogeneous split root systems than by exposing whole roots to specific levels of NaCl or at least comparing the salt effect in the two different situations (Rahnama et al., 2011; Bazihizina et al., 2012; Kong et al., 2012).

Since it is unquestionable that the elucidation of fundamental molecular and physiological responses to salinity is instrumental to improving crops salt tolerance, in the present study uniform and non-uniform salinity have been simulated with a split-root system in which the root system was divided into two equal portions and each portion irrigated with 0 mM (control) or 100 mM NaCl (salt stress) solution and 10 mM KNO_3_. Moreover, for the uniform salinity treatment (with the entire root system grown at 0 or 100 mM NaCl), low and high nitrate concentrations (0.1 and 10 mM KNO_3_, respectively) are applied.

These conditions are used to study physiological root responses to salinity focusing on: (i) root ions accumulation and effect on some physiological parameters; (ii) osmolytes accumulation and contribution with ions to the osmotic balance of the root cells; (iii) expression and activity of the main enzymes involved in the synthesis of nitrogen-containing osmolytes; (iv) antioxidant response.

## Materials and methods

### Plant material and growth conditions

Seeds of durum wheat (*Triticum durum* Desf. cv. Ofanto) were supplied from the Center for Cereal Research of Foggia (Italy) and germinated in the dark on filter paper moistened with deionized water at 21°C. Thereafter, individual seedlings were transferred to 4.5 L pots placed into a phytotron under controlled conditions (16 h photoperiod, 350 μmol m^−2^ s^−1^ PAR, thermoperiod 25:20°C day:night, 65% relative humidity). Initially the pots contained distilled water, that was replaced after 3 days with a modified (nitrogen-free) Hoagland medium (Carillo et al., 2005), and then after other 3 days with Hoagland medium containing 0.1 or 10 mM KNO_3_. The nutrient solution was continually aerated and replaced every 3 days.

Starting from day 10 of hydroponic culture, the medium was supplemented with 50 mM NaCl, increased to 100 mM NaCl 1 day later. The gradual exposure of plants to the increasing NaCl reflected that of field growing conditions, and prevented salt shock (Woodrow et al., 2016). A subgroup of 10 mM KNO_3_ grown plants was cultured in a split-root system with half of their roots treated with or without 100 mM NaCl. The control plants in the other pot from each group were grown without supplemented NaCl. The root length of six replicate plants of each treatment on days 5, 10, 15, and 20 of hydroponic culture was measured. The roots of 20-day-old plants were immediately used for the determination of physiological and morphological parameters or stored at −80°C.

### Physiological and morphological measurements

Roots were immediately weighed to obtain the fresh weight and re-weighted after floating on deionized water for 24 h at 4°C in the dark and after being dried at 70°C for 48 h. The relative water content (RWC) was obtained as [(root fresh weight − root dry weight)/(root turgor weight − root dry weight)] × 100. Water potential was measured by using a pressure bomb (Scholander et al., 1965). The root vigor index (RVI) was calculated as: RVI = percentage germination (~88%) × average roots dry weight (in mg) (Woodrow et al., 2016).

For light microscopy fresh root were cut in 2 mm or smaller size pieces with a razor blade with the aid of a stereomicroscope. Samples were placed on a glass slide in water, covered with a cover slip and immediately examined. Microscopy was performed on an Olympus BX51 microscope (Olympus Optical Co., Hamburg, Germany) equipped with differential interference contrast (DIC). Roots were examined at 20, 40, and 100 magnification; for this latter a 100 oil-immersion objective was used. Images were captured using a digital camera and CellPrism software.

### Ions, osmolality, hydrogen peroxide, and metabolites analysis

Ions were assayed according to Carillo et al. (2011). Root sap osmolality was measured according to Cuin et al. (2009). The amounts of hydrogen peroxide (H_2_O_2_) were determined according to Baptista et al. (2007). Total proteins, starch and sugars were evaluated according to Carillo et al. (2012). Total fructans were measured according to Morcuende et al. (2004). Fructan classes were determined according to Cimini et al. (2015). Starch and total fructans were expressed as glucose equivalents.

Primary amino acids, proline, and glycine betaine were extracted and assayed according to Woodrow et al. (2016). Ascorbic acid (ASCAc), dehydroascorbic acid (DHA), reduced and oxidized glutathione (GSH and GSSG) were extracted as described by Annunziata et al. (2012) and Woodrow et al. (2012) and determined according to Queval and Noctor (2007). Malondialdehyde was assayed according to Carillo et al. (2011). Contribution of metabolites and ions to osmolality was calculated according to Cuin et al. (2009) and Puniran-Hartley et al. (2014).

### Enzyme extractions and assays

All the procedures for root enzyme extractions and assays were carried out at 4°C. Enzymes were extracted according to Gibon et al. (2004), except where differently indicated. Asparagine synthetase (AS; EC 6.3.5.4) was extracted in roots of 20-day-old plants and immediately desalted and assayed in a solution containing 1 mM aspartate semialdehyde (an inhibitor of asparaginase) and 1 mM amino(oxy)acetic acid (an inhibitor of aspartate aminotransferase) according to Duff et al. (2011). NADH-dependent glutamate synthase (Fd-GOGAT; EC 1.4.1.14), glutamine synthetase (GS; EC 6.3.1.2), and nitrate reductase (NR; EC 1.6.6.1) were assayed according to Gibon et al. (2004). Phosphoenolpyruvate carboxylase (PEPC; EC 4.1.1.31) was assayed according to Esposito et al. (1998). Deaminating glutamate dehydrogenase activities (GDH; EC 1.4.1.2) was determined according to Skopelitis et al. (2007). Δ1-pyrroline-5-carboxylate synthetase activity (P5CS; EC 2.7.2.11) was determined according to Parre et al. (2010). For all assayed enzyme activities, parallel control experiments were performed after desalting the extracts via centrifugal filtration through Sephadex G-25 PD-10 columns (Amersham Biosciences) equilibrated with Hepes-KOH 50 mM pH 7.5, MgCl_2_ 10 mM, dithiothreitol 1 mM and eluted by spinning at 1800 g for 1 min. The enzyme activities were expressed as μmol h^−1^ g^−1^ FW.

### RNA extraction and cDNA synthesis

Total RNA was isolated from powdered roots according to Woodrow et al. (2016). RNA quantity and quality were determined spectrophotometrically using the NanoDrop ND-1000 UV-VIS (Thermo Scientific, Wilmington, MA) and separated on 1.5% agarose gel stained with SYBR safe (Invitrogen). mRNA was purified from ~500 μg of total RNA using a mRNA Isolation Kit (Roche) following manufacturer's instructions. First strand cDNA was synthesized from 1 μg of mRNA by reverse transcriptase with both random hexamer primers and anchored oligo dT according to the instructions of the SensiFAST^™^ cDNA Synthesis Kit (Bioline).

### RT-PCR and gene expression

Asparagine synthetase (*Asn1, Asn2, Asn3*), Δ-pyrroline-5-carboxylate synthetase (*P5CS*), and nitrate reductase (*NR*) gene expression analysis was carried out by semiquantitative RT-PCR reactions, using Transcriptor High Fidelity cDNA Synthesis Sample Kit (Roche). RT-PCR was performed in a total volume of 50 μl containing 300 ng of the first strand cDNA reaction products, 5 μl of FastStart Buffer with 20 mM MgCl_2_, 0.2 mM deoxynucleotides, 50 pmol of primers (Table S1), and 2 U of FastStart Taq DNA polymerase (Roche). RT-PCR analysis was performed using gene-specific primers for *Asn1, Asn2*, and *Asn3* isoforms (Wang et al., 2005; Gao et al., 2016), *NR* (Carillo et al., 2005; Wang et al., 2005), and degenerate primers for *P5CS* (Woodrow et al., 2016) (Table S1). The amount of *TdAsn1, TdAsn2, TdAsn3, TdNR*, and *TdP5CS* templates mRNA levels were based on the comparison with the level of the 190 bp mRNA for actin (Woodrow et al., 2010), a constitutively expressed “house-keeping” gene. The semi-quantitative PCR was used to estimate the transcript levels. All PCR reactions included an initial denaturation step of 2 min at 95°C. Afterwards, in order to prevent amplifications reaching the plateau phase, several dilution tests (1:5; 1:10; 1:15) were performed combined with various numbers of cycles (30–35) with a denaturation step (30 s at 95°C), an annealing step (30 s at 40–70°C), an extension step (2 min at 72°C), and a final extension for 7 min at 72°C. Finally the experiments with a 1:5 dilution and 35 cycles were carried out. Amplification products were visualized on 1.5% (w/v) agarose gels, using a UV light. Densitometric evaluation of DNA bands was performed with the Imager 1D/2D software (Image Lab v. 3.0, Bio-Rad). Band intensity was expressed as relative absorbance units. Band signals were normalized using the actin signals.

### Cloning and sequencing of P5CS cDNA

The 0.5 kb *P5CS* cDNA amplification products were purified from agarose gel and cloned into a pGEM-T Easy Vector system II (Promega) by mixing 2 μL of amplified product with 25 ng of pGEM-T Easy Vector, 3 U T4 ligase, and 1 μL ligation buffer in 10 μL volume. The ligation product was cleaned with sec-butanol and precipitated with ethanol. The sample was resuspended in 10 μL of 0.5 M Tris-EDTA and transformed into *Escherichia coli* cells. Twenty clones were sequenced by BMR Genomics (Padova).

### Statistical analysis

Roots from six plants for each treatment were used for determination of length, measurements of fresh and dry weight, and water potential. The other analyses were performed on four biological replicates for each treatment. The analysis of variance (ANOVA) and the Pearson correlation analysis were performed by SigmaPlot 12 software (Systat Software Inc., Richmond, CA, USA). The mean differences were compared to their corresponding Least Significant Differences (LSD) at 0.05 and 0.01 confidence levels. A heat map generated in Excel (Carillo et al., 2008) was used to summarize the plant responses to the salt and light stresses. Results were calculated as log_2_ (salt stress or HL values/average of controls) and were visualized using a false color scale, with blue indicating an increase and red a decrease of values relative to those in control condition. No differences were visualized by white squares. Principal component analysis (PCA) on the different analyzed parameters was carried out using Multibase 2015, an Excel add-in program for Windows (http://www.numericaldynamics.com) according to Ciarmiello et al. (2015).

## Results

### Root growth and physiological parameters

**Figure 1:**
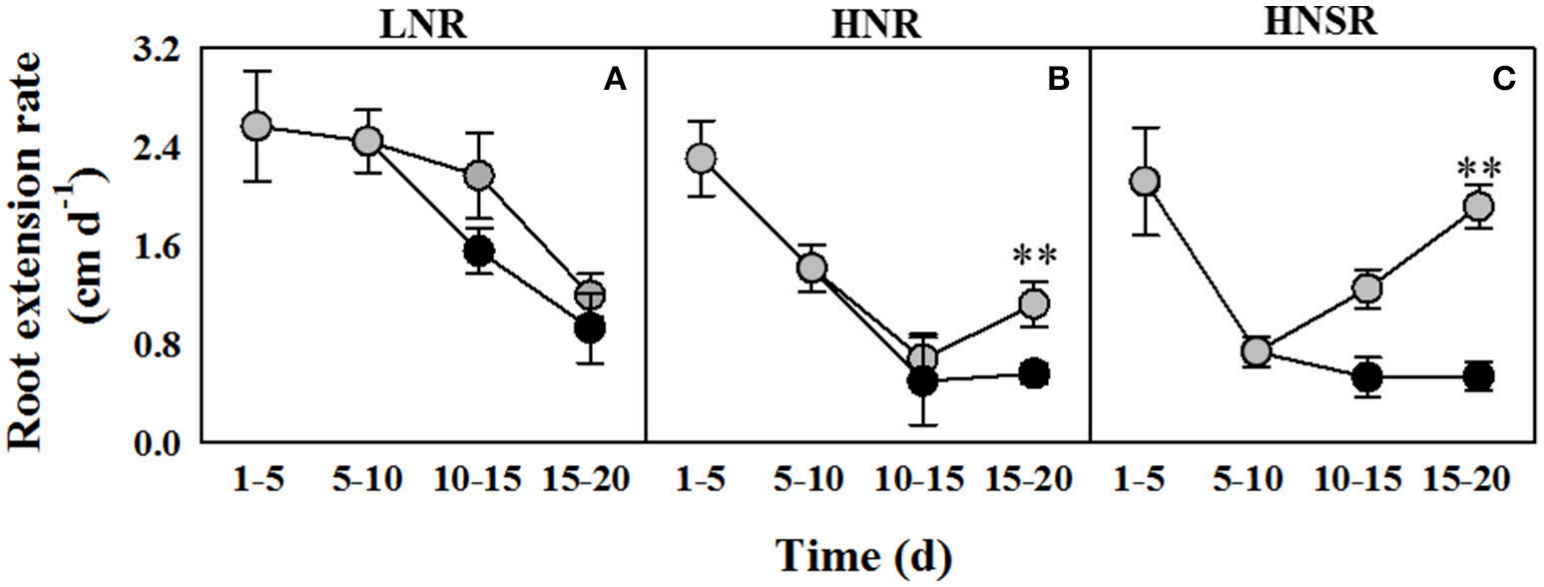
**Root extension rate of durum wheat roots cultured in 0.1 (LNR, A)** and 10 mM ${\text{KNO}}_{3}^{-}$ with (HNR, **B**) or without (HNSR, **C**) split root system, under control (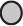) or salt (100 mM NaCl, 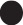) conditions. Six replicate plants of each treatment were measured on days 5, 10, 15, and 20 of hydroponic culture. ${\text{KNO}}_{3}^{-}$ was added on day 5 and 100 mM NaCl was added from day 10. The values are means ± *SD* (*n* = 6). Significant differences between treatments are indicated by asterisks (^**^p < 0.01; LSD-test).

The extension rate of roots of wheat seedlings at low nitrate (LNR) was higher than that of high nitrate roots (HNR) and high nitrate split roots (HNSR) between day 10 and 15 either in control and salt stressed plants (Figures 1A–C). Between day 15 and 20 in plants under salinity the extension rate of HNR and HNSR strongly increased compared to LNR, and in particular in HNSR it was significantly higher (*P* < 0.01) than in the other two treatments (Figure 1C). Nonetheless, at day 20 the length of HNR and HNSR was significantly lower (*P* < 0.05) than LNR either under control or salinity treatment (Table 1). The fresh weight of HNR was 1.4-fold higher than that of LNR, independently of salinity. The fresh weight of HNSR in control conditions did not differ significantly from HNR one, while that of HNSR under salinity was about 3-fold smaller than that of salt stressed HNR (Table 1). The root dry weight, showed a different pattern, being similar in control and in salt stressed treatments (on average 48.8 or 45.6 mg per plant, respectively), independently of nitrogen treatment, with the exception of HNSR under salinity that showed the lowest weight (14.6 mg per plant) (Table 1).

**Table 1:** **Physiological parameters, ions and hydrogen peroxide, carbohydrates, MDA, ascorbic acid, and glutathione expressed per g fresh weight in roots of durum wheat seedlings grown with 0.1 or 10 mM ${\text{NO}}_{3}^{-}$ (with or without root split system), under 0 or 100 mM NaCl**.

	**0.1 mM ${\text{NO}}_{3}^{-}$**		**10 mM ${\text{NO}}_{3}^{-}$**		**10 mM ${\text{NO}}_{3}^{-}$ split**	
	**0 mM NaCl**	**100 mM NaCl**	**0 mM NaCl**	**100 mM NaCl**	**0 mM NaCl**	**100 mM NaCl**
**PHYSIOLOGICAL PARAMETERS**						
Lenght (cm)	66.1 ± 9.4^a^	41.5 ± 3.7^b^	28.3 ± 5.3^cd^	22.8 ± 2.5^c^	34.0 ± 1.4^d^	24.0 ± 3.0^c^
Fresh weight (mg/plant)	544 ± 64^a^	402 ± 34^b^	750 ± 63^c^	544 ± 31^a^	639 ± 98^c^	139 ± 16^d^
Dry weight (mg/plant)	49.3 ± 3.7^a^	42.8 ± 1.6^b^	51.1 ± 4.3^a^	48.1 ± 4.6^ab^	46.0 ± 4.4^ab^	14.6 ± 1.0^c^
RWC	92.0 ± 4.2^ac^	43.4 ± 8.2^b^	96.9 ± 3.4^a^	88.9 ± 1.3^c^	94.0 ± 0.5^a^	85.8 ± 5.5^c^
Root vigor index	43.4 ± 3.0^ac^	37.7 ± 1.04^b^	45.0 ± 2.2^a^	42.3 ± 1.7^ac^	40.5 ± 1.2^c^	13 ± 0.7^d^
Root water potential (MPa)	−0.40 ± 0.09^a^	−0.58 ± 0.08^b^	−0.25 ± 0.04^c^	−0.39 ± 0.03^a^	−0.30 ± 0.06^ac^	−0.56 ± 0.05^b^
Sap osmolality (mOsmol kg^−1^)	343 ± 36^a^	575 ± 55^b^	284 ± 47^c^	543 ± 61^b^	289 ± 33^ac^	470 ± 55^b^
Root/Shoot DW ratio	1.06 ± 0.13^a^	1.45 ± 0.16^b^	0.55 ± 0.04^c^	0.59 ± 0.07^c^		
**IONS AND HYDROGEN PEROXIDE (**μ**mol g**^−1^ **FW)**						
Chloride	30.3 ± 2.7^a^	111.8 ± 13^b^	23.6 ± 2.0^c^	62.7 ± 7.5^d^	20.9 ± 3.1^c^	59.7 ± 1.6^c^
Nitrate	0.05 ± 0.01^a^	0.10 ± 0.01^a^	34.5 ± 4.8^b^	23.4 ± 4.9^c^	35.4 ± 0.6^d^	19.3 ± 0.5^c^
Potassium	119 ± 13^a^	87.3 ± 6.4^b^	79.1 ± 15.8^b^	113 ± 9.1^a^	86.0 ± 8.3^b^	98.0 ± 10.0^b^
Sodium	19.2 ± 1.72^a^	101.3 ± 14^b^	10.5 ± 1.1^c^	84.7 ± 16.1^b^	14.1 ± 2.6^c^	77.9 ± 15.0^b^
Potassium:Sodium	6.22 ± 0.70^a^	0.86 ± 0.11^b^	7.51 ± 0.62^a^	1.33 ± 0.14^c^	6.10 ± 0.80^a^	1.26 ± 0.15^c^
Hydrogen peroxide	1.79 ± 0.21^a^	4.76 ± 0.61^b^	1.18 ± 0.24^c^	3.16 ± 0.35^d^	1.99 ± 0.31^a^	3.26 ± 0.84^d^
**CARBOHYDRATES (**μ**mol g**^−1^ **FW)**						
Starch (Geq)	15.6 ± 2.6^a^	14.4 ± 3.5^a^	13.0 ± 1.7^a^	14.0 ± 1.54^a^	13.0 ± 1.4^a^	14.3 ± 1.6^a^
Hexoses	8.83 ± 0.72^a^	5.60 ± 0.41^b^	6.06 ± 0.87^b^	5.55 ± 0.20^b^	6.04 ± 0.61^b^	4.66 ± 0.32^c^
Sucrose	5.11 ± 0.31^a^	8.60 ± 0.91^b^	4.33 ± 0.83^a^	4.90 ± 0.36^a^	6.17 ± 0.70^a^	5.86 ± 0.84^a^
Total fructans (G_eq_)	44.1 ± 5.3^a^	30.9 ± 1.7^b^	9.13 ± 3.27^c^	18.6 ± 2.8^d^	11.9 ± 2.5^c^	14.5 ± 1.7^cd^
1-Kestose	5.69 ± 0.27^a^	4.85 ± 0.32^b^	0.36 ± 0.03^c^	0.89 ± 0.12^d^	0.46 ± 0.07^c^	0.72 ± 0.09^d^
Inulin	0.38 ± 0.06^a^	0.63 ± 0.10^b^	0.20 ± 0.03^c^	0.158 ± 0.02^c^	0.28 ± 0.02^d^	0.10 ± 0.02^e^
Nystose	1.216 ± 0.088^a^	6.024 ± 1.253^b^	0.533 ± 0.045^c^	1.341 ± 0.10^a^	0.67 ± 0.09^c^	1.10 ± 0.14^a^
1-Fructofuranosylnystose	2.925 ± 0.36^a^	4.948 ± 0.292^b^	1.652 ± 0.269^ac^	2.013 ± 0.40^a^	2.04 ± 0.19^a^	1.52 ± 0.18^c^
Total fructans:Starch	2.83 ± 0.33^a^	2.14 ± 0.24^b^	0.70 ± 0.09^c^	1.33 ± 0.11^d^	0.92 ± 0.13^c^	1.01 ± 0.16^c^
**MDA, ASCORBIC ACID, AND GLUTATHIONE (nmol g**^−1^ **FW)**						
MDA	6.51 ± 1.58^a^	12.43 ± 0.89^b^	18.2 ± 1.8^c^	11.7 ± 1.3^b^	16.6 ± 1.2^c^	14.5 ± 2.9^bc^
AsAc	0.66 ± 0.11^a^	0.63 ± 0.12^a^	0.64 ± 0.1^a^	0.77 ± 0.17^a^	0.53 ± 0.08^a^	0.85 ± 0.13^b^
DHA	0.62 ± 0.04^a^	0.29 ± 0.05^b^	0.64 ± 0.08^a^	2.45 ± 0.36^c^	0.69 ± 0.09^a^	1.91 ± 0.24^c^
AsAc +DHA	1.27 ± 0.10^a^	0.92 ± 0.14^b^	1.29 ± 0.20^a^	3.22 ± 0.42^c^	1.22 ± 0.15^a^	2.76 ± 0.31^b^
DHA:AsAc	0.96 ± 0.22^a^	0.49 ± 0.12^b^	1.00 ± 0.15^a^	3.17 ± 0.41^c^	1.30 ± 0.22^a^	2.25 ± 0.35^d^
GSH	20.2 ± 1.6^a^	4.0 ± 0.3^b^	67.8 ± 5.4^c^	70.3 ± 5.6^c^	44.7 ± 3.6^d^	86.6 ± 8.9^e^
GSSG	40.8 ± 3.5^ad^	9.5 ± 2.0^b^	21.3 ± 1.7^c^	50.2 ± 6.2^a^	38.4 ± 3.7^d^	14.6 ± 1.3^e^
GSH + GSSG	60.9 ± 4.9^a^	13.5 ± 1.1^b^	89.1 ± 7.1^c^	120 ± 11^d^	83.1 ± 6.7^c^	101 ± 8^d^
GSSG:GSH	2.02 ± 0.11^a^	2.39 ± 0.26^a^	0.31 ± 0.07^b^	0.71 ± 0.06^c^	0.86 ± 0.10^c^	0.17 ± 0.01^d^

*Values are mean ± SD (n = 4). Means in the same row with different letters are significantly different (p < 0.05, LSD-test). On the right a heat map summarizes the differences between samples*.

The RWC of control LNR and HNR and HNSR was about 92, 97, and 94%, respectively. Salinity halved the RWC in LNR and decreased of about 8% that of HNR and HNSR (Table 1).

The root vigor index (RVI) was, on average, 43 in control roots both at low and high nitrate. The RVI decreased by 22, 30, and 70% of controls in LNR, HNR, and HNSR under salinity, respectively (Table 1).

The root water potential (Y_w_) was higher in control than in salt stressed plants. Salinity reduced it from −0.40 and −0.25 MPa of control LNR and HNR, respectively, to values of about −0.58 and −0.39. HNSR showed root Y_w_ similar to that of LNR either in control and salt stress treatment (Table 1).

### Ions and hydrogen peroxide content

The concentration of chloride (Cl^−^) and sodium (Na^+^) in roots of either control and salt stressed plants decreased when nitrate (${\text{NO}}_{3}^{-}$) concentration in the culture medium increased, even though not significantly for Na^+^ under salinity, while significantly for Cl^−^. This latter was about 30 and 22 μmol g^−1^ FW in control LNR and HNR and HNSR and 112 and 61 μmol g^−1^ FW in salt stressed LNR and HNR and HNSR, respectively (Table 1).

The Na^+^ content was 19.2 ± 1.7 and 101 ± 14 μmol g^−1^ FW in control and salt stressed LNR, and 10.5 ± 1.1 and 84.7 ± 16.1 μmol g^−1^ FW in control and salt stressed oHNR, respectively. The Na^+^ content of HNSR was similar to that of HNR (Table 1).

The ${\text{NO}}_{3}^{-}$ concentration of roots at low nitrate was similar to that of the nutrient solution, while in HNR and HNSR at 10 mM KNO_3_ it exceeded that of the nutrient solution by about 3.5- and 2.1-fold in control and salt stressed plants, respectively (Table 1).

Potassium (K^+^) content ranged between about 87 and 110 μmol g^−1^ FW and was not significantly dependent on ${\text{NO}}_{3}^{-}$ or salt treatment (Table 1). The K^+^ to Na^+^ content ratio, which provides information about the potential of the plants to discriminate the two ions (Gorham et al., 1990), was, on average, 6.7 in all control roots. This value was significantly decreased (*p* < 0.01) by the salt treatment to 0.86 and about 1.3 in salt treated LNR and HNR, respectively (Table 1).

The hydrogen peroxide concentration of roots was 1.9 μmol g–1 FW in control LNR and HNSR and 1.2 μmol g–1 FW in control HNR. In response to salinity, its content increased 2.7-fold in LNR and HNR and 1.6-fold in HNSR (Table 1).

### N-containing compounds

The total proteins of control roots of LNR and HNR and HNSR were, on average, 3.3 mg g^−1^ FW. Salinity did not significantly change their content (Table 2).

**Table 2:** **Total proteins, free amino acids, glycine betaine (GB), and enzyme activities in durum wheat roots under 0.1 and 10 mM ${\text{NO}}_{3}^{-}$ (with or without root split system), under 0 and 100 mM NaCl**.

	**0.1 mM ${\text{NO}}_{3}^{-}$**		**10 mM ${\text{NO}}_{3}^{-}$**		**10 mM ${\text{NO}}_{3}^{-}$ split**	
	**0 mM NaCl**	**100 mM NaCl**	**0 mM NaCl**	**100 mM NaCl**	**0 mM NaCl**	**100 mM NaCl**
Total proteins (mg g^−1^ FW)	2.93 ± 0.42^a^	3.11 ± 0.52^ab^	3.45 ± 0.36^b^	2.90 ± 0.21^a^	3.62 ± 0.29^b^	3.11 ± 0.44^ab^
**AMINO ACIDS AND GB (**μ**mol g**^−1^ **FW)**						
Total free amino acids	2.57 ± 0.14^a^	3.85 ± 0.22^b^	8.14 ± 0.86^c^	11.3 ± 0.8^d^	10.1 ± 0.9^cd^	9.22 ± 0.66^c^
Alanine	0.11 ± 0.01^a^	0.31 ± 0.01^b^	0.75 ± 0.06^c^	0.77 ± 0.08^c^	0.59 ± 0.00^d^	0.63 ± 0.06^cd^
Arginine	0.04 ± 0.00^a^	0.06 ± 0.00^b^	0.06 ± 0.01^b^	0.09 ± 0.01^c^	0.07 ± 0.01^b^	0.06 ± 0.01^b^
Asparagine	0.11 ± 0.01^a^	0.31 ± 0.02^b^	0.27 ± 0.05^b^	2.45 ± 0.23^c^	0.72 ± 0.06^d^	0.75 ± 0.11^d^
Aspartate	0.13 ± 0.01^a^	0.32 ± 0.01^b^	1.23 ± 0.13^c^	1.32 ± 0.12^c^	1.48 ± 0.03^d^	0.96 ± 0.08^e^
Cysteine	0.03 ± 0.00^a^	0.04 ± 0.00^b^	0.04 ± 0.00^b^	0.05 ± 0.00^c^	0.06 ± 0.01^cd^	0.07 ± 0.01^d^
Glutamine	0.28 ± 0.06^a^	0.27 ± 0.03^a^	0.71 ± 0.13^b^	1.05 ± 0.08^c^	1.51 ± 0.01^d^	1.69 ± 0.06^e^
Glutamate	0.56 ± 0.03^a^	0.94 ± 0.07^b^	3.26 ± 0.48^c^	2.22 ± 0.23^d^	3.20 ± 0.02^c^	2.45 ± 0.19^d^
Glycine	0.04 ± 0.00^a^	0.04 ± 0.00^a^	0.06 ± 0.01^b^	0.06 ± 0.00^b^	0.07 ± 0.01^bc^	0.09 ± 0.01^c^
Histidine	0.08 ± 0.01^a^	0.09 ± 0.01^a^	0.13 ± 0.02^b^	0.24 ± 0.02^c^	0.16 ± 0.00^d^	0.13 ± 0.01^b^
Isoleucine	0.11 ± 0.01^a^	0.10 ± 0.01^a^	0.07 ± 0.00^b^	0.13 ± 0.01^c^	0.08 ± 0.00^b^	0.06 ± 0.00^d^
Leucine	0.13 ± 0.01^a^	0.11 ± 0.01^ab^	0.10 ± 0.01^b^	0.13 ± 0.01^a^	0.10 ± 0.00^b^	0.07 ± 0.01^c^
Lysine	0.07 ± 0.00^a^	0.09 ± 0.01^b^	0.03 ± 0.01^c^	0.07 ± 0.01^a^	0.05 ± 0.01^d^	0.08 ± 0.01^b^
Metionine	0.03 ± 0.00^a^	0.04 ± 0.00^a^	0.05 ± 0.00^b^	0.05 ± 0.00^b^	0.06 ± 0.00^b^	0.05 ± 0.00^b^
Phenilalanine	0.03 ± 0.00^a^	0.09 ± 0.00^b^	0.19 ± 0.06^c^	0.07 ± 0.01^bc^	0.08 ± 0.01^b^	0.06 ± 0.00^c^
Proline	0.41 ± 0.03^a^	0.54 ± 0.02^b^	0.55 ± 0.06^b^	1.50 ± 0.10^c^	0.83 ± 0.11^d^	1.10 ± 0.06^e^
Serine	0.13 ± 0.01^a^	0.18 ± 0.00^b^	0.28 ± 0.02^c^	0.39 ± 0.04^d^	0.32 ± 0.01^c^	0.41 ± 0.03^d^
Threonine	0.09 ± 0.02^a^	0.09 ± 0.01^a^	0.16 ± 0.03^b^	0.20 ± 0.02^b^	0.20 ± 0.02^b^	0.17 ± 0.01^b^
Triptophane	0.06 ± 0.00^a^	0.07 ± 0.02^a^	0.03 ± 0.00^b^	0.05 ± 0.00^a^	0.03 ± 0.00^b^	0.02 ± 0.00^b^
Tyrosine	0.02 ± 0.00^a^	0.02 ± 0.00^a^	0.05 ± 0.01^b^	0.26 ± 0.02^c^	0.28 ± 0.04^c^	0.24 ± 0.02^c^
Valine	0.11 ± 0.01^a^	0.13 ± 0.01^a^	0.13 ± 0.02^a^	0.21 ± 0.02^b^	0.17 ± 0.01^c^	0.13 ± 0.01^a^
Glutamine:Glutamate	0.50 ± 0.01^a^	0.29 ± 0.03^b^	0.22 ± 0.03^c^	0.47 ± 0.07^a^	0.47 ± 0.04^a^	0.69 ± 0.09^d^
Amides	0.39 ± 0.02^a^	0.58 ± 0.04^b^	0.98 ± 0.07^c^	3.50 ± 0.18^d^	2.24 ± 0.18^e^	2.44 ± 0.03^e^
Minor amino acids	0.69 ± 0.06^a^	0.80 ± 0.10^a^	0.83 ± 0.08^a^	1.29 ± 0.11^b^	1.10 ± 0.08^bc^	0.90 ± 0.09^c^
BCAAs	0.36 ± 0.04^a^	0.34 ± 0.03^a^	0.30 ± 0.02^a^	0.47 ± 0.04^b^	0.35 ± 0.03^a^	0.25 ± 0.02^c^
GB	0.86 ± 0.13^a^	2.58 ± 0.30^b^	0.67 ± 0.05^a^	2.43 ± 0.36^b^	0.70 ± 0.11^a^	0.64 ± 0.19^a^
**ENZYME ACTIVITIES (**μ**mol h**^−1^ **mg**^−1^ **PROT)**						
AS	0.38 ± 0.02^a^	0.66 ± 0.10^b^	0.73 ± 0.04^b^	1.67 ± 0.20^c^	1.02 ± 0.15^d^	0.96 ± 0.12^d^
GDH	6.54 ± 0.82^a^	5.54 ± 0.29^a^	4.50 ± 0.48^a^	4.79 ± 0.32^a^	4.03 ± 0.58^a^	4.41 ± 0.36^a^
GOGAT	2.99 ± 0.30^a^	1.04 ± 0.11^b^	2.38 ± 0.36^a^	4.71 ± 0.65^d^	2.58 ± 0.18^a^	2.53 ± 0.29^a^
GS	18.1 ± 1.7^ac^	19.6 ± 1.1^a^	9.82 ± 0.75^b^	15.5 ± 1.4^c^	9.49 ± 1.05^b^	11.1 ± 1.3^b^
NR	5.81 ± 0.61^a^	3.77 ± 0.36^b^	11.2 ± 1.87^c^	9.43 ± 1.56^c^	9.40 ± 1.51^c^	8.57 ± 0.66^c^
NiR	55.2 ± 4.7^a^	31.9 ± 4.7^b^	63.6 ± 2.8^c^	46.9 ± 4.5^ad^	53.6 ± 4.4^a^	41.1 ± 2.7^d^
P5CS	1.99 ± 0.31^a^	2.06 ± 0.19^a^	2.16 ± 0.23^a^	4.50 ± 0.33^b^	3.35 ± 0.42^c^	3.46 ± 0.27^c^
PEPC	1.45 ± 0.22^a^	0.64 ± 0.09^b^	2.08 ± 0.17^c^	1.39 ± 0.13^a^	1.59 ± 0.15^a^	1.26 ± 0.18^a^

*Values are mean ± SD (n = 4). Means in the same row with different letters are significantly different (p < 0.05, LSD-test)*.

The total free amino acid concentration of roots of control plants depended on nitrate nutrition, and was 3.6-fold higher in HNR than in LNR (Table 2). Glutamate, proline, glutamine, aspartate, and asparagine were quantitatively the major amino acids representing about 58, 74, and 77% of total free amino acids in LN, HN, and HNs control roots, respectively (Table 2). Salinity significantly increased the free amino acid concentration in LNR and HNR (1.5- and 1.4-fold, respectively, *p* < 0.01), but not in HNSR. This result was mostly due to alanine, asparagine, and aspartate which increased 2.8-, 2.8- and 2.5-fold, respectively, in LNR, and to asparagine and proline which increased 9- and 2.7-fold, respectively, in HNR (Table 2). While the slight decrease of free amino acids in HNSR under salinity was mostly due to aspartate and glutamate which decreased by 35 and 24%, respectively (Table 2). Glutamate content was decreased by salinity by 32% in HNR, while increased by it (1.7-fold) in LNR (Table 2, *p* < 0.01).

Salinity increased the glutamine to glutamate ratio by 2.2- and 1.5-fold in HNR and HNSR, but reduced it by 42% in LNR compared to respective controls (Table 2).

Salinity increased the minor amino acids content in HNR by 1.6-fold compared to respective control, and this increase was mainly due to tyrosine and branched chain amino acids (BCCAs) which increased 5.7- and 1.6-fold, respectively (Table 2).

The glycine betaine (GB) concentration in roots was highly dependent on salinity except for HNSR, being, on average, 0.8 and 2.5 μmol g^−1^ FW in control and salt stressed treatments of LNR and HNR, independently of nitrogen nutrition. HNSR showed a constant value of GB similar to that of LNR and HNR control plants either in control and salt treated plants (Table 2).

### Carbohydrates content

Starch content, not significantly affected by nitrate nutrition and salinity in roots, was, on average, 14 μmol G g^−1^ FW (Table 1).

Sucrose concentration was, on average, 5.2 μmol g^−1^ FW in all control roots and in salt treated HNR and HNSR, while salinity increased its content by 1.7-fold in LNR (Table 1). Root hexose content (glucose and fructose) was 8.83 ± 14 μmol g^−1^ FW in control LNR, while it significantly decreased (*p* < 0.05) in all other treatments (on average, −37%) and in particular in HNSR in which it almost halved (Table 1). Fructans content was 44.1 and 10.5 μmol g^−1^ FW in control LNR and HNR and HNSR, respectively (Table 3). Salinity significantly decreased total fructans (−30%, *p* < 0.01) in LNR, while doubled them in HNR. The total fructans to starch ratio had a similar trend to fructans (Table 1). Among root fructans, nystose (GF_4_), 1-Fructofuranosylnystose (GF_4_), and inulin (GF29 dahlia type) increased under salinity of 5-, 1.7-, and 1.7-fold in control LNR, respectively; while under salt stress only nystose was significantly increased (*p* > 0.05) of 2.5- and 1.6- in HNR and HNSR, respectively, but remaining at a concentration about 5-fold lower than that found in LNR under salinity (Table 1).

**Table 3:** **Relative contribution (%) of inorganic ions, amino acids, glycine betaine, sucrose, fructans, and other metabolites toward the total osmolality**.

	**cLNR**	**sLNR**	**cHNR**	**sHNR**	**cHNRS**	**sHNSR**
Chloride	8.84	19.43	8.33	11.5	7.24	12.7
Nitrate	0.01	0.02	12.2	4.32	12.2	4.12
Potassium	34.7	15.2	27.9	20.8	29.8	20.9
Sodium	5.59	17.6	3.71	15.6	4.88	16.6
Ions contribution	49.2	52.3	52.0	52.3	54.1	54.2
Total amino acids	0.75	0.67	2.87	2.08	3.49	1.96
Asparagine	0.03	0.05	0.10	0.45	0.25	0.16
Glutamine	0.08	0.05	0.25	0.19	0.52	0.36
Minor AA	0.10	0.06	0.11	0.09	0.12	0.05
Proline	0.12	0.09	0.19	0.28	0.29	0.23
Glycine betaine	0.25	0.45	0.24	0.45	0.24	0.14
Hexoses	2.57	0.97	2.13	1.02	2.09	0.99
Sucrose	1.49	1.50	1.52	0.90	2.14	1.25
Kestose	1.66	0.84	0.13	0.16	0.16	0.15
Inulin	0.11	0.11	0.07	0.03	0.10	0.02
Nistose	0.35	1.05	0.19	0.25	0.23	0.23
Fructofuranosyl nystose	0.85	0.86	0.58	0.37	0.71	0.32
Organic osmolytes	8.04	6.45	7.73	5.26	9.15	5.07
Other metabolites	42.8	41.3	40.2	42.5	36.7	40.7

*Values are mean ± SD (n = 4). The SD was lower than 13% of the average value*.

### Malondialdehyde, ascorbic acid, and glutathione

Malondialdehyde (MDA) levels were significantly higher (*p* < 0.01) in salt stress LNR compared to control ones. In HNR MDA content was higher in control roots than in salt treated ones, while HNSR shower a similar value that was, on average, 15.5 nmol g^−1^ FW (Table 1).

Ascorbic acid (AsAc) level (Table 1) was, on average, 0.6 nmol g^−1^ FW in LNR and HNR, independently of salinity, and in control HNSR. Salt stress HNSR showed an AsAc content 1.3-fold higher than in the all other treatments. Dehydroascorbic acid (DHA) was at the same concentration as GSH in control roots. Salinity almost halved DHA in LNR, but strongly increased it in HNR and HNSR by 3.8- and 2.8-fold, respectively. The ratio of DHA/AsAc showed a similar trend to DHA (Table 1).

Reduced glutathione (GSH) content was dependent on nitrogen nutrition and salinity. In LNR, the GSH in control plants was at the highest concentration in the HNR (67.8 nmol g^−1^ FW) compared with LNR (20.2 nmol g^−1^ FW) and HNSR (44.7 nmol g^−1^ FW). GSH content was decreased by salt treatment in LNR (−80%) while almost doubled in HNSR. Oxidized GSH (GSSG) had a concentration of about 40 nmol g^−1^ FW in LNR and HNSR and of 21.3 nmol g^−1^ FW in HNR under control conditions. Salinity increased GSSG in HNR by 2.4-fold (*p* < 0.01), but it strongly decreased its content in the other treatments. The GSSG/GSH ratio was significantly higher (*p* < 0.01) in control LNR, independently of salinity, compared to the other treatments (Table 1).

### Ions and metabolites contribution to the root osmolality

Durum wheat sap osmolality was, on average, 305 mOsmol kg^−1^ in all control roots, while it significantly increased (*P* < 0.01) after salinity treatment, reaching a value about 1.7-fold higher than that of the respective controls (Table 1). The relative contribution of the inorganic ions to osmolality was on average 51.8 and 52.9% for control and salt stressed roots, respectively (Table 3). In particular, the relative ion contribution toward osmolality increased from 8.8 to 19.4% for chloride and from 5.6 to 17.6% for sodium in LNR; while it varied from about 7.8–21.1% for chloride and from 4.3 to 16.1% for sodium in HNR and HNSR. On the contrary, the potassium contribution toward osmolality decreased under salinity from 34.7 to 15.2% in LNR, and from about 28.8–20.8% in HNR and HNSR. Only in HNR and HNSR, nitrate contribution toward osmolality decreased under salinity from 12.2 to about 4.2%. It is interesting to note that the contribution of nitrate and potassium together to osmolality was of about 40% in controls while it decreased to 15 and 25% in LNR and HN(S)R under salinity, respectively (Table 3).

The contribution of the measured organic osmolytes to osmolality was, on average, 8.3 and 5.6% in control and salt stressed roots, respectively. It was due for about 85 and 55% to sugars in LNR and HN(S)R, respectively (Table 3).

According to Puniran-Hartley et al. (2014), it is possible to speculate that other metabolites present in the cell can contribute to osmolality, and their relative contribution can be calculated as difference between the total sap osmolality (Table 1) and the contribution of the measured major inorganic ions and organic osmolytes shown in Table 3. The calculated organic osmolytes contribution was therefore 50.8 and 48% in control LNR and HNR, 47.7% in salt stressed LNR and HNR, and 45.8% for HNSR, independently of salinity (Table 3).

### Ions and metabolites expressed in terms of dry weight

Given the salt stress induced-reduction of RWC in LNR and of dry weight in HNSR, ions and metabolites results were also expressed in terms of dry weight (Tables S2, S3). However, while the data about LNR and HNR were in agreement with those reported in literature, keeping almost unchanged the salt stressed values to control values ratio (with fluctuations of <10%), the amount of metabolites and ions in the salt stressed HNSR samples were so concentrated to appear unlikely (about 3-fold higher than other data previously reported). This finding made difficult to carry out a proper effective comparison between the concentrations of ions and metabolites when values were expressed on a dry weight basis. In particular, the increase of ions and metabolites concentrations coincided with a 4.6- and 3.1-fold decrease of salt-treated HNSR fresh and dry weight compared with the respective controls (Tables S2, S3).

### Gene expression

**Figure 2:**
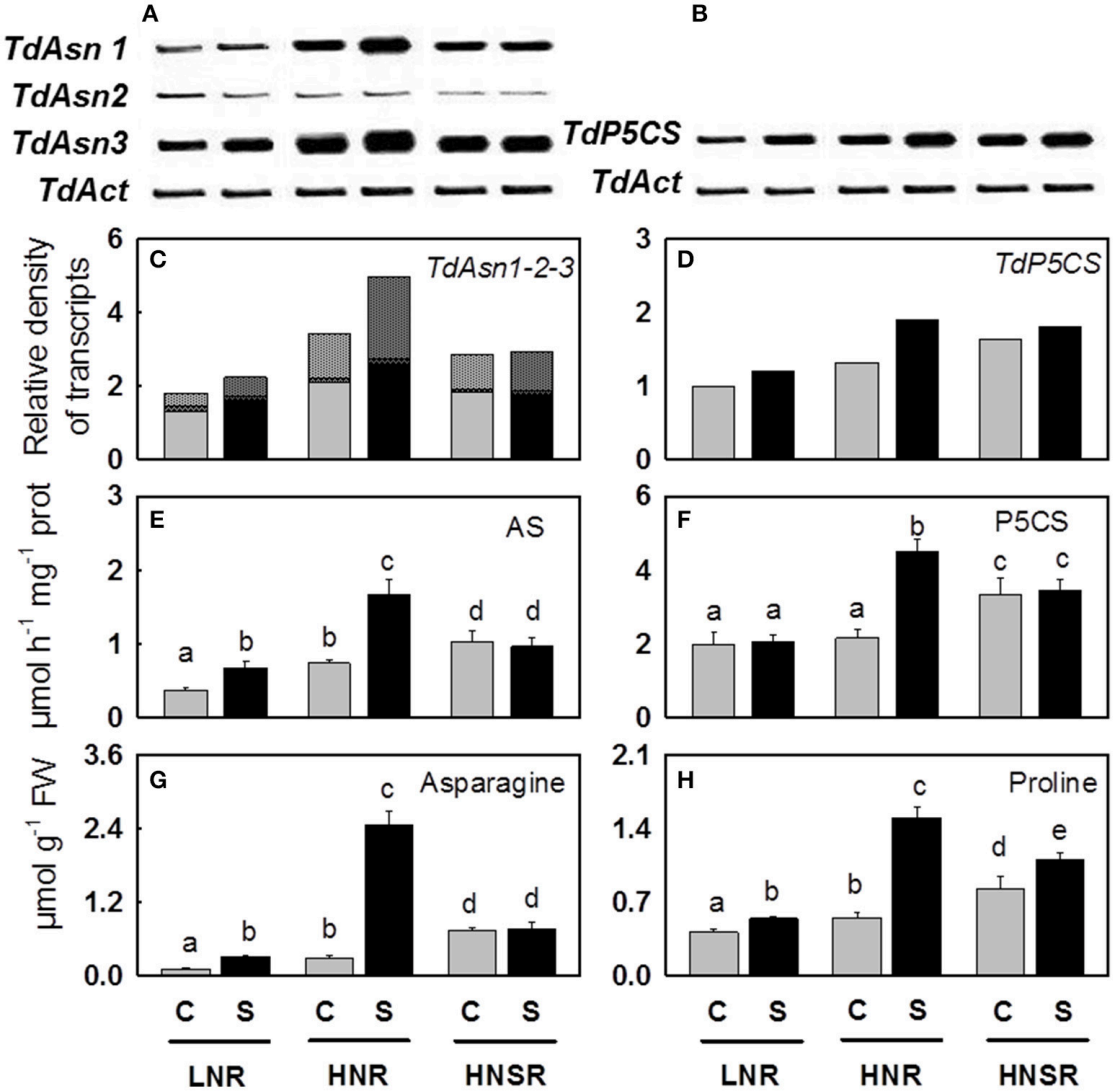
**Asparagine synthetase (***Asn1***, ***Asn2***, ***and Asn3***) (A)** and Δ1-pyrroline-5-carboxylate synthase (*P5CS*) **(B)** genes and their relative densitometric quantification normalized using the actin signals **(C,D)**, the AS **(E)** and P5CS **(F)** enzymatic activities, the content of asparagine **(G)**, and proline **(H)** in roots of control (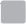) and salt stressed (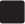) plants. Plants were subjected to salt stress starting from day 10 of culture. Control plants were grown without NaCl addition. Plants were harvested after 20 days of hydroponic culture. The values are mean ± *SD* (*n* = 4). Different letters above bars indicate significant difference between treatments (*p* < 0.05, LSD-test).

Nitrate reductase (*NR*), asparagine synthetase (*Asn1, Asn2, Asn3*) and Δ1-pyrroline-5-carboxylate synthase (*P5CS*) genes showed differential expression levels (Figure 2 and Figure S1).

Nitrogen increased the expression level of *TdNR*, being the highest expression level found in HNSR independently of salinity (Figure S1).

Three out of four isoforms of *Asn* present in wheat (Gao et al., 2016) were considered (*TdAsn1, TdAsn2, and TdAsn3*), using three different primer pairs, because they can be up-regulated by nitrogen and/or salt stress (Wang et al., 2005; Antunes et al., 2008; Gao et al., 2016). The three isoforms were expressed in all treatments. *TdAsn1* and *TdAsn3* expression was highly inducible by nitrogen and salinity; it was detected at higher extent in HNR and HNSR. While *TdAsn2* was expressed at very low level in all treatments (Figures 2A,C and Figure S1).

Using degenerate primers (formed of a mix of four different combinations) we found an unique *TdP5CS* transcript significantly up-regulated by salt stress independently of nitrogen treatment (*p* < 0.01), even mainly expressed in HNR and HNSR. In order to understand if also for *P5CS* different isoforms are present in durum wheat, the PCR products were cloned and the sequenced cDNA clones were used as a query in a BLASTN search for all wheat A and B chromosomes with the GrainGenes 2.0 database (http://wheat.pw.usda.gov/GG2/index.shtml) and URGI database (https://wheat-urgi.versailles.inra.fr/) (Barabaschi et al., 2015). *T. durum* is, in fact, an allotetraploid plant with a AABB genome (2*n* = 4*x* = 28) formed through hybridization between two separate but related diploid species, *T. monococcum* or *T. urartu* (AA, 2*n* = 14) and *T. searsii* or *T. speltoides* (BB, 2*n* = 14). Search results showed an identity of 98–100% with the *P5CS* transcripts belonging to *T. durum* cv. Strongfield, *T. durum* Cappelli, *T. urartu*, and *A. speltoideas* plants. The alignment of the 20 *P5CS* transcript sequences obtained (Figure S4) revealed two or three single point mutations among clones, generating six different fragments of similar size. These latter showed a high homology with four nucleotide sequences identified on chromosomes 1B, 3A, 3B (in two different locus) and 7A, according to Mayer et al. (2014) which found that A and B sub-genomes contain very similar proportions of genes (60.1–61.3%). The six different transcripts were *P5CS* orthologs and paralogs (Wang et al., 2014).

### Enzyme activities

Nitrate reductase (NR) activity was dependent on nitrate nutrition (Table 2). The NR activity was 5.8 ± 0.6 and 11.2 ± 1.9 μmol ${\text{NO}}_{2}^{-}$ h^−1^ mg^−1^ protein, respectively. Salt treatment significantly reduced the NR activity only in LNR (−35%, *p* < 0.01). The activation state of NR in control roots was about 90%, independently of nitrogen nutrition and salt treatment (Carillo et al., 2005).

Nitrite reductase (NiR) activity was between 4.8- and 9.5-fold higher than the NR activity in the same treatments. In particular NiR activity was about 54 μmol h^−1^ mg^−1^ protein in LNR and HNR and HNSR and 64 μmol h^−1^ mg^−1^ protein in HNR. Salinity decreased it by 42% in LNR and by about 23% in HNR and HNSR (Table 2).

Glutamine synthetase (GS) activity, due only to the cytosolic GS isoforms in durum wheat roots (Nigro et al., 2016), was, on average, 18.9 μmol h^−1^ mg^−1^ protein in control and salt stressed LNR. GS activity significantly decreased in control HNR and control and salt treated HNSR (−46%, *p* < 0.01), while it remained unvaried in salt treated HNR (Table 2).

Glutamate synthase (GOGAT) showed a similar activity in control LNR and HNR and HNSR, that was, on average, 2.6 μmol h^−1^ mg^−1^ protein. Salinity significantly increased GOGAT activity by 2-fold (*p* < 0.01) only in HNR, while significantly decreased it in LNR (–65%, *p* < 0.01; Table 2).

Deaminating glutamate dehydrogenase (GDH) activity was, on average, 6.0 and 4.4 μmol h^−1^ mg^1^ protein in LNR and HNR and HNSR, respectively, independently of salinity (Table 2).

In response to salinity, AS activity increased of 1.8 and 2.3 in LNR and HNR compared to the respective controls, reaching 0.66 and 1.67 μmol h^−1^ mg^−1^ protein, while it did not significantly vary (*p* > 0.05) in HNSR independently of salinity (Table 2; Figure 2E).

Phosphoenolpyruvate carboxylase (PEPC) activity was about 1.5 μmol h^−1^ mg^−1^ protein in LNR and HNSR and 2.1 μmol h^−1^ mg^−1^ protein in HNR. Salinity significantly decreased it in LNR (−56%) and HNR (–33%) (*p* < 0.01; Table 2).

Δ1-pyrroline-5-carboxylate synthetase (P5CS) activity was, on average, 2.9 μmol h^−1^ mg^−1^ protein in control and salt stressed LNR and control HNR, and 4.8 μmol h^−1^ mg^−1^ protein in HNSR. Salinity increased significantly HNR activity by 2.1-fold (Table 2, *p* < 0.01).

### Microscopy of root tips

Root tips of durum wheat plants were observed by DIC microscopy (**Figure 4**). In Figure 1A, a salt stressed root tip was divided in three zones pointing out the meristem (1), elongation (2), and mature cells (3). The root tips from control plants were characterized by densely packed tissues with small intercellular spaces (**Figure 4B**). Root tips from salt stressed plants showed extensive vacuolization and lack of typical organization of apical tissue; moreover a slight plasmolysis due to a lack of continuity and adherence between cells was present with a tendency to the arrest of growth and differentiation (**Figure 4C**). At higher magnification the presence of salt crystals between the wall and the cell membrane, and in vacuoles (though smaller) and plastids were observed (**Figure 4D**). The lack of cuticle in the roots allowed to exclude the silicon nature of these aggregates. These latter were not visible in control root tips (not shown).

### Statistical analysis

The principal component analysis (PCA) of all analyzed parameters expressed for fresh weight showed a well-defined separation among samples from the different treatments. The first two principal components accounted for 63.5% of the variation. The PCA scatter-plot split the samples into five main groups. Nitrogen nutrition contributed to the clear separation on component 1 (PC1), which described 42% of the variability, while salinity contributed to separation on PC2, which described 21.5% of the variability (Figure S2A). In the Figure S2B only the top 10 contributors were highlighted. In particular, asparagine, proline and minor amino acids highly influenced the salt stressed HNR and HNSR samples grouped in the first quadrant. GB, fructans to starch ratio and sucrose influenced the salt stressed LNR samples in the second quadrant. While potassium to sodium ratio and root Y_w_ influenced control HNR, and nitrate and MDA influenced control HNSR both present in the fourth quadrant.

In the Figure S3, the PCA of all analyzed parameters expressed for dry weight showed an unforeseen separation among salt stressed HNSR samples from all other different treatments. The first two principal components accounted for 82.7% of the variation. The PCA scatter-plot split the samples into three main groups. Salt stressed HNSR clustered on the border between the fourth and the first quadrant, fully separated from all other HN(S)R samples grouped in the second quadrant, and the LNR samples present in the third quadrant (Figure S3).

**Figure 3:**
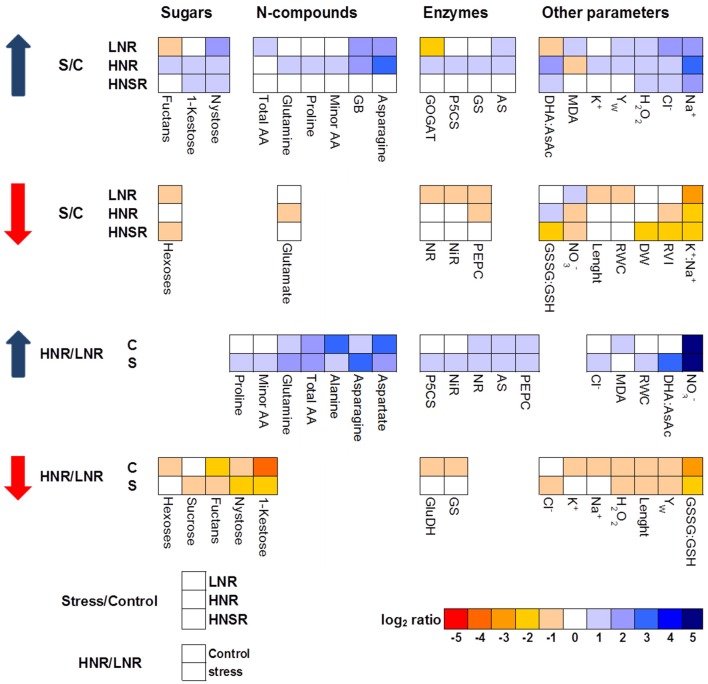
**Heat map analysis summarizing the plant responses to nitrogen nutrition and salinity**. Results were calculated as Logarithm base 2 (Log_2_) of salt stressed values/control values (S/C) or high nitrogen/low nitrogen (HN/LN) for all the treatments, LN, HN, and HN_split_ (LNR, HNR, and HNSR). Results were visualized using a false color scale, with blue indicating an increase and red a decrease of values relative to those in control condition. No differences were visualized by white squares.

A heat map representing the changes in metabolite levels in shoots under different treatments provided an integrated view of the effect of nitrogen nutrition and salinity on durum wheat roots (Figure 3). The most interesting result was the strong increase induced by salinity of GB and sucrose in LNR and of GB, asparagine and proline in HNR. While no differences were found between control and salt stressed HNSR.

## Discussion

Durum wheat, as other plants, displays elaborate root plastic responses to a heterogeneous environment such as soil, in which nutrients and salts concentration in the circulating solution can be extremely spatially and temporally variable, actively prioritizing growth toward nutrients, or trying to limit its exposure to salinity (Bazihizina et al., 2012; Galvan-Ampudia et al., 2013; Nacry et al., 2013; Kiba and Krapp, 2016; Koevoets et al., 2016).

Durum wheat root extension was, in fact, highly induced by N limitation (Table 1) as already found in a wide range of species, including maize and Arabidopsis (Roycewicz and Malamy, 2012). Moreover, nitrogen affects the distribution of sugars across plant organs (Lemoine et al., 2013), and in particular N-limitation determined an accumulation of carbohydrates in LNR which was correlated with higher root growth rate and root/shoot ratio (Marschner, 1986; Stitt et al., 2002; Remans et al., 2006).

**Figure 4:**
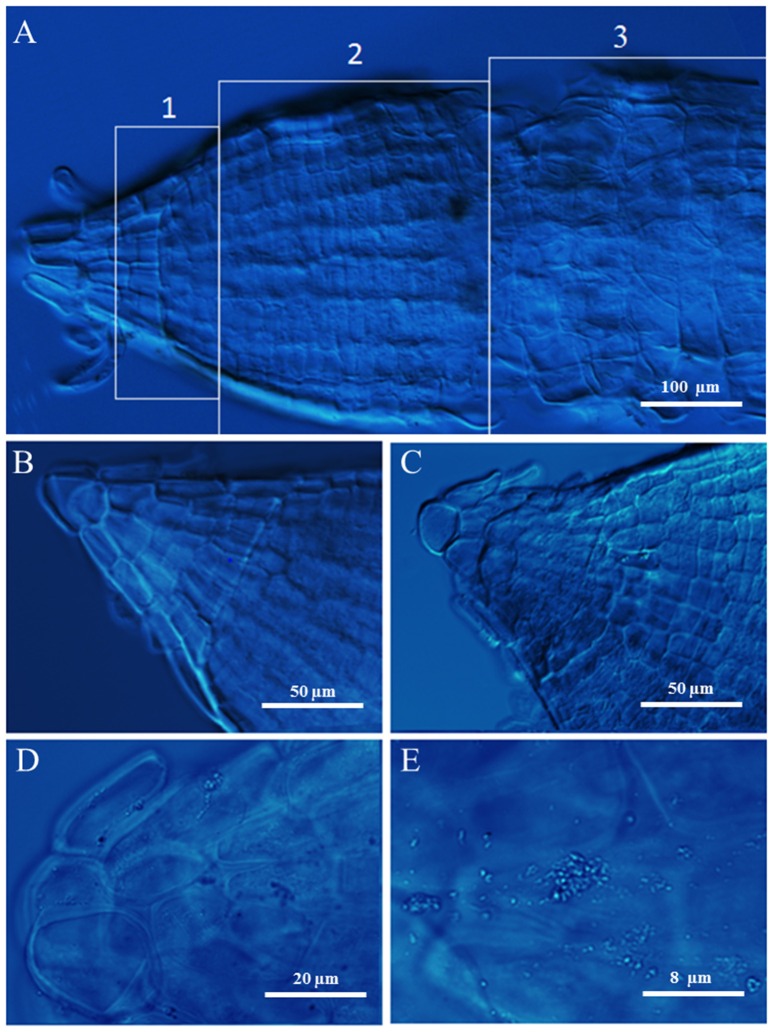
**Root tips of durum wheat grown in absence (B)** or presence of 100 mM NaCl **(A,C–E)** observed using DIC microscopy. In A the numbers 1, 2, and 3 point out the meristem, elongation, and mature root zones, respectively.

Since nitrogen is acquired entirely by the root system, the increase in the root/shoot ratio allows plants to have more chances to obtain nitrogen to sustain growth (Roycewicz and Malamy, 2012). This is in agreement with the fact that plants, in response to a shortage in mineral nutrition, allocate more resources to the organs involved in mineral acquisition, for increasing root surface and allowing a more efficient exploitation of nutrients in relation to their spatial distribution in the soil (Stitt, 1999; Stitt et al., 2002; Zhang and Pilbeam, 2011). Moreover, high level of nitrate in the medium resulted in a higher root weight but a decrease of root length and root/shoot ratio, probably dependent on the accumulation of nitrate itself in the plant (Stitt, 1999; Roycewicz and Malamy, 2012) (Table 1). On the contrary, salinity and, even more, N-limitation and salinity reduced not only durum wheat extension rate and consequently root length, but also fresh and dry weight as well as root vigor index in agreement with earlier studies (Neumann et al., 1994). Reduction in root extension rates might come from the marked lowering of root turgor and water potential (Rodriguez et al., 1997), as also reported in other species (Qin et al., 2010). However, Neumann et al. (1994) reported that lower root extension in maize could be related to hardening of cell walls and not to changes in water potential. Indeed, in wheat roots the apical zone showed signs of wall hardening, with a tendency to the arrest of growth and differentiation (Figure 4).

Partial salt stress applied through split-root system affected at the highest extent the part of the root exposed to salt. The strong reduction of HNSR length and weight in the fraction of root treated with salinity vs. the significant increase of control HNSR ones is in agreement with the compensatory growth described in non-stressed areas of root systems under stress (Schumacher and Smucker, 1984). Plants with split roots probably make the salt stressed part stop growing because there is the other root part that can work for the uptake of water and nutrients. This allows the salt stressed HNSR to accumulate large amounts of organic compounds by spending all the available energy (also that needed for growth) without jeopardizing shoot growth and survival.

Whereas, the better conditions of salt stressed HNR compared to LNR ones can be explained by the ability of these plants to realize an osmotic adjustment that maintains a high RWC even at low root water potential, resulting in maintenance of turgor and prevention of tissue desiccation (Morgan, 1984). Osmotic adjustment helps cells to withstand salt stress by maintaining sufficient turgor for growth and metabolism to proceed and involve transport, accumulation, and compartmentation of inorganic ions and compatible solutes (Munns, 2002; Carillo et al., 2008, 2011; Wu et al., 2015).

The increase of salinity not only increased the absolute value for chloride and sodium in the sap, but also the relative contribution of these ions to osmolality (Puniran-Hartley et al., 2014), being the total contribution almost stable. In fact, since the synthesis of osmolytes has a huge cost (50–70 moles ATP for mole; Raven, 1985; Shabala, 2013), it is highly unlikely that the cell could adjust the ion balance only by increasing *de novo* synthesis of compatible metabolites (Shabala, 2013). On the contrary, the increase in root sodium and chloride content suggested that durum wheat cells could use these ions as a cheap osmoticum for turgor maintenance by sequestering them in vacuoles (Puniran-Hartley et al., 2014). At the same time the osmolarity of cytosol was matched with that of vacuole by the reshaping of few classes of metabolites (N-containing ones and sugars) used for multiple purposes, that is as osmolytes and for protection against oxidative stress. Indeed, an ~100 mOsm increase in organic osmolytes level between control and salt stressed roots (Table 1) is enough to osmotic balance the root cell, assuming most of them are located in the cytosol and that the cytosol represents <10% of the root cell total volume fraction (Cuin et al., 2009), while vacuoles and apoplast occupy almost 85 and 5% of it, respectively (Munnich and Zoglauer, 1979; Lee et al., 1990; Patel et al., 1990; Rodriguez et al., 1997).

GB, one of the main nitrogen-containing compatible osmolytes found in durum wheat under salt stress (Carillo et al., 2005; Ashraf and Foolad, 2007; Carillo et al., 2008), was accumulated at the same extent in LNR and HNR under salinity being independent of nitrate treatment, but dependent on salinity (Figure 1A). The lack of an influence of N nutrition on GB accumulation in roots suggests that organic N reserves within the plant can be mobilized to satisfy the demand resulting from salt stress (Carillo et al., 2008). GB and sucrose, but not proline, played a major role during osmotic adjustment of LNR under salinity (Figure 3 and Figure S2).

In HNR proline contribution to the osmotic adjustment increased while that of sucrose decreased (Table 3). A strong correlation was found among proline content, P5CS activity, *TdP5CS* transcript (*r* ≥ 0.94; *P* < 0.001). This result suggested that at high nitrate salt stress can induce *TdP5CS* gene expression and activity causing a *de novo* synthesis and accumulation of proline according to (Strizhov et al., 1997; Carillo et al., 2008). Moreover, the presence of *P5CS* orthologs and paralogs could satisfy the need for high amounts of proline or provide an efficient means for a differential transcriptional regulation in response to stress (Long and Dawid, 1980; Rai and Penna, 2013).

Fructans, which were highly concentrated in low nitrate treatment independently of salinity, increased in HNR under salinity compared to the respective control while the other carbohydrates remained constant (Table 1). One of the advantages of accumulation of fructans in the protection against abiotic stress is the high water solubility of these carbohydrates (Livingston et al., 2009). Accumulation of fructans can contribute to membrane stabilization (Valluru and Van den Ende, 2008) and, even indirectly, to the release of sugars which can take part in osmotic adjustment reducing the cytosol water potential and allowing root cell expansion under salt stress (Krasensky and Jonak, 2012).

Glutamate and glutamine highly increased in control HNR. Their increase in roots in presence of nitrate as nitrogen source is supported by several studies and explained by the nitrate induction of the GS-GOGAT pathway specifically localized in the proplastids of roots. This latter is a pathway not available for ammonium assimilation in the absence of nitrate (Britto and Kronzucker, 2002). Glutamate decreased in HNR under salinity according to Woodrow et al. (2016) (Table 2). The decrease of glutamate could depend on its use as nitrogen donor in biosynthetic transamination for the production of amides, in particular asparagine which strongly increased in salt stressed HNR (Forde and Lea, 2007) (Table 2, Figures 2G, 3). The increase of asparagine was probably due to a *de novo* synthesis catalyzed by the isoforms of asparagine synthetase *TdAsn1* and *TdAsn3*, which were strongly induced by simultaneous salinity and high levels of nitrogen metabolites (e.g., glutamine and glutamate; Lam et al., 1994; Wang et al., 2005; Lea et al., 2007; Gao et al., 2016). In particular, a very strong correlation was found between *TdAsn1*, AS, and asparagine (*r* ≥ 0.92; *P* < 0.001). The up-regulation of AS genes by salt and other abiotic stresses were also reported in maize and Arabidopsis (Chevalier et al., 1996; Wong et al., 2004).

The increase of asparagine, as well as glutamine, has been previously reported in wheat leaves (Carillo et al., 2005; Wang et al., 2005), as well as its possible role in osmotic adjustment, macromolecule protection and ammonium detoxification (Herrera-Rodríguez et al., 2004). Minor amino acids significantly increased only in salt treated HNR, potentially functioning both as compatible compounds and antioxidant (Woodrow et al., 2016). Their variations can depend on an increase of carbohydrates and/or amides (Noctor et al., 2002; Fritz et al., 2006) or on changes of glutamine to glutamate ratio (Table 2).

The significant increase of total amino acids in salt stressed HNR was not replicated in HNSR in the same conditions (Table 2).

However, the total contribution of GB, amino acids and soluble sugars to osmolality in root tissues was quite low, about 5.6% in all roots under salinity. This means that different compounds accounting for about 41.5% of total osmolality, were accumulated in roots and participated to the osmotic balance and oxidative stress protection of root cells under salt stress.

Total ascorbate (AsAc) and glutathione (GSH), which are of paramount importance in the prevention or repair of damages deriving from ROS (Noctor and Foyer, 1998), increased only in salt stressed HNR and HNSR, as well as GSSG to GSH ratio and DHA to AscAc ratio, with the exception of GSSG to GSH ratio in salt treated HNSR (Table 1). This indicates that the ASC–GSH cycle did not play a crucial role for scavenging ROS especially under simultaneous high nitrate and salinity condition.

In LNR, MDA, a marker of lipid peroxidation and therefore, indirectly, of cell damage, was significantly increased under salt stress treatments and was well-correlated with hydrogen peroxide accumulation (Table 1). Unexpectedly, high levels of MDA were found in control HNR and HNSR where low levels of hydrogen peroxide were present. Schmid-Siegert et al. (2012) has reported that MDA in roots cannot derive from lipid peroxidation of polyunsaturated fatty acids. The aldehyde is pathogen-inducible in these regions and its level can be increased by cellular mediators that are involved both in defense and growth.

## Conclusions

Durum wheat roots under salinity showed few changes in selected metabolites which allowed the plant viability even at low nitrate. This metabolic rearrangement was necessary to meet the demand for anti-stress agents including compatible solutes and antioxidants (Obata and Fernie, 2012). Thus, while the sodium was used as osmoticum in the vacuole, mainly glycine betaine, sucrose, nystose, and 1-fructofuranosylnystose at low nitrate, and glycine betaine, asparagine and proline at high nitrate were responsible for the osmotic adjustment, the assimilation of the excess of ammonium and the scavenging of ROS under salinity in the cytosol. The strong increase of the sole asparagine and glutamine in HNSR, either in control and salt stress conditions, suggests that the stress-induced adjustment is not a regional effect. On the contrary, the plant operates as an integrated system in which metabolic stress-induced signals spread in the plant and change the metabolism even in areas in which the stress conditions are not present. Notwithstanding this, different parts of plant root systems may behave as physiologically autonomous units, differing their responses to environmental signals (Gašparíková et al., 2002), and preserving their own capability to supply the shoots with water, nutrients or assimilates (Shani et al., 1993). In this way one part of the root system can compensate the plant for a decreased supply or a loss of functionality by the other part, optimizing plant viability under heterogeneous water, nutrients or stressing conditions (Shani et al., 1993).

## Author contributions

PC designed the research; MA, LC, PW, EM, and PC performed the research; PC and AF analyzed the data; PC wrote the paper. All authors read and approved the final manuscript.

## Funding

MA and EM thank Max Planck Society for funding. LC, PW, AF, and PC thank Seconda Universitá degli Studi di Napoli and Campania Region (Italy) PSR 214-f2 action within the project “Network for the protection and management of genetic resources, agrofood (AGRIGENET).”

### Conflict of interest statement

The authors declare that the research was conducted in the absence of any commercial or financial relationships that could be construed as a potential conflict of interest.
