# Supplementary material for: Durum Wheat Roots Adapt to Salinity Remodeling the Cellular Content of Nitrogen Metabolites and Sucrose
Source: Front Plant Sci. 2017 Jan 9;7:2035. doi: 10.3389/fpls.2016.02035 (PMC5220018; doi:10.3389/fpls.2016.02035)

**Supplemental table 2.** Ions and hydrogen peroxide, carbohydrates, MDA, ascorbic acid and glutathione expressed per g dry weight in roots of durum wheat seedlings grown with 0.1 or 10 mM NO_3_^-^ (with or without root split system), under 0 or 100 mM NaCl . Values are mean ± SD (n=4). Means in the same row with different letters are significantly different (p<0.05, LSD test). On the right a heat map summarises the plant responses to nitrogen nutrition and salinity. Results were calculated as Logarithm base 2 (Log_2_) of salt stressed values/control values (S/C), high nitrogen /low nitrogen (HN/LN) or HN_split_/HN (HNSR/HNR). Results were visualized using a false colour scale, with blue indicating an increase and red a decrease of values relative to those in control condition. No differences were visualized by white squares.


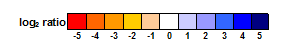

Supplement: Supplementary file 2 [file Table2.DOCX]
