# Supplementary material for: Durum Wheat Roots Adapt to Salinity Remodeling the Cellular Content of Nitrogen Metabolites and Sucrose
Source: Front Plant Sci. 2017 Jan 9;7:2035. doi: 10.3389/fpls.2016.02035 (PMC5220018; doi:10.3389/fpls.2016.02035)
Supplement: Supplementary file 4 [file DataSheet1.DOCX]

**SUPPLEMENTAL FIGURE 1** *T. durum* asparagine synthetase (*Asn1, Asn2 and Asn3*), Δ1-pyrroline-5-carboxylate synthase (*P5CS*), nitrate reductase (*NR*) and actin (*Act*) genes.

**SUPPLEMENTAL FIGURE 2** Principal component analysis (PCA) scatter plots based on the first two principal components (PC1 and PC2) generated for the different treatments and all the analysed physiological parameters, metabolites and enzyme activities expressed for fresh weight (A), and the top ten parameters which contributed most to the segregation of the different treatments (B).

**SUPPLEMENTAL FIGURE 3** Principal component analysis (PCA) scatter plots based on the first two principal components (PC1 and PC2) generated for the different treatments and all the analysed physiological parameters, metabolites and enzyme activities expressed for dry weight

**SUPPLEMENTAL FIGURE 4** Multiple sequence alignment of *P5CS* cDNAs. Names of sequences are listed on the left. ‘*’ indicates nucleotides identical in the same position.
