# Supplementary figures and images for: Durum Wheat Roots Adapt to Salinity Remodeling the Cellular Content of Nitrogen Metabolites and Sucrose
Source: Front Plant Sci. 2017 Jan 9;7:2035. doi: 10.3389/fpls.2016.02035 (PMC5220018; doi:10.3389/fpls.2016.02035)

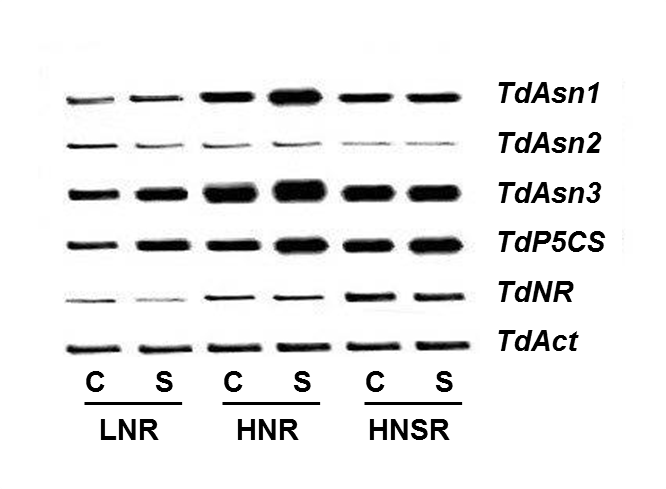

Supplement: Supplementary file 5 [file Image1.TIF]

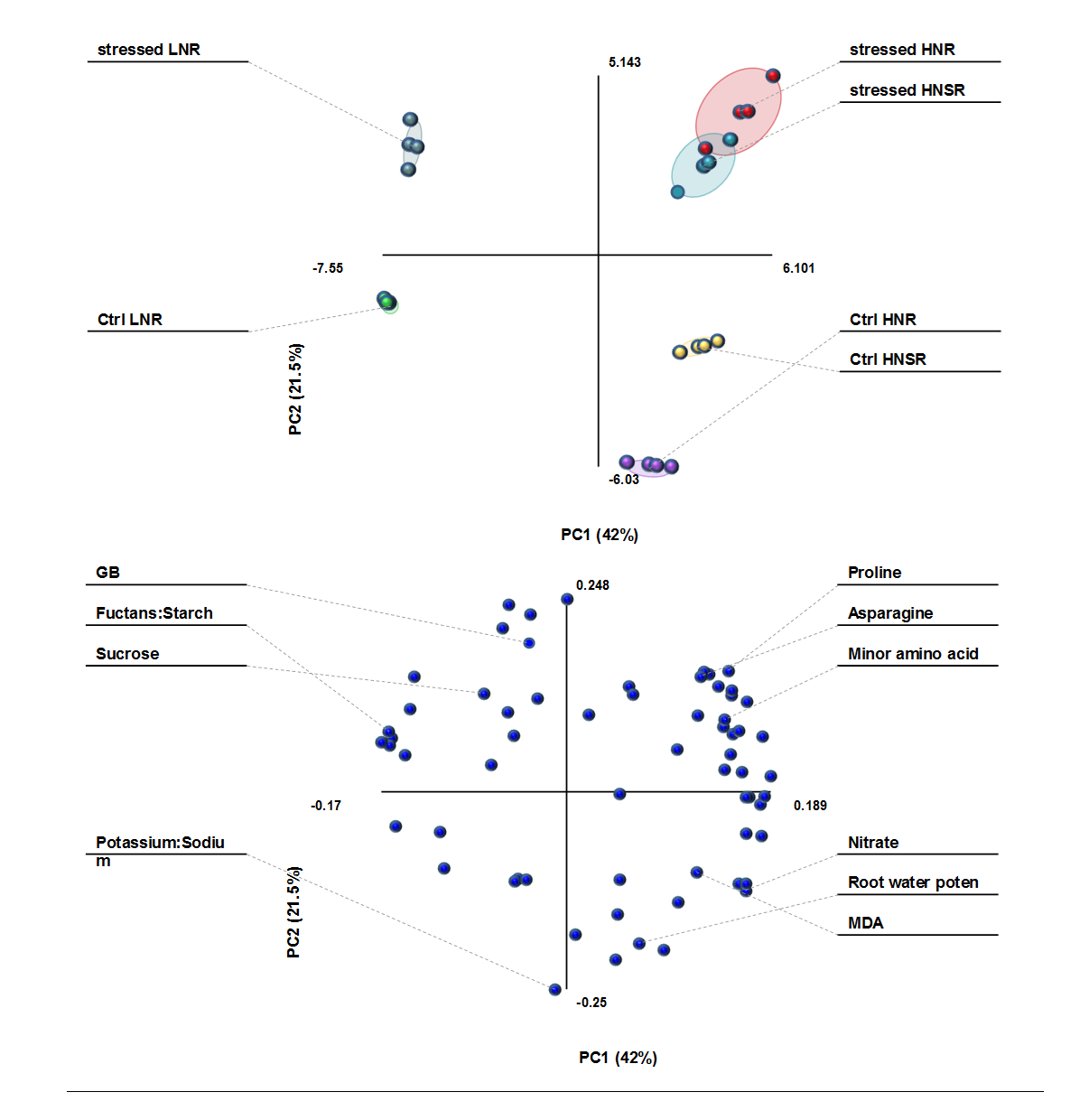

Supplement: Supplementary file 6 [file Image2.TIF]

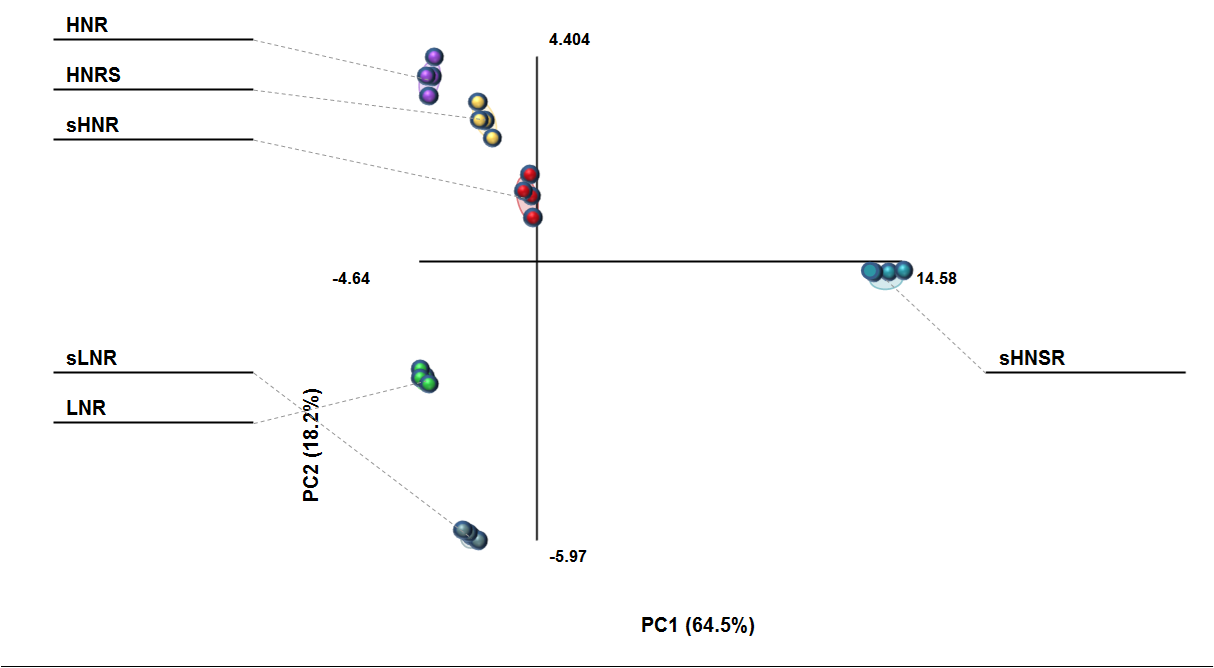

Supplement: Supplementary file 7 [file Image3.TIF]
