# Supplementary material for: Durum Wheat Roots Adapt to Salinity Remodeling the Cellular Content of Nitrogen Metabolites and Sucrose
Source: Front Plant Sci. 2017 Jan 9;7:2035. doi: 10.3389/fpls.2016.02035 (PMC5220018; doi:10.3389/fpls.2016.02035)
Supplement: Supplementary file 8 [file Image4.PDF]

```
26P5CS          CTGGTATATTCTGGGATAACGACAGTTTGGCGACGCTGTTGGCAAAAGAACTGGATGCTG
28P5CS          CTGGTATATTCTGGGATAACGACAGTTTGGCGACGCTGTTGGCAAAAGAACTGGATGCTG
19P5CS          CTGGTATATTCTGGGATAACGACAGTTTGGCGACGCTGTTGGCAAAAGAACTGGATGCTG
17P5CS          CTGGTATATTCTGGGATAACGACAGTTTGGCGACGCTGTTGGCAAAAGAACTGGATGCTG
12P5CS          CTGGTATATTCTGGGATAACGACAGTTTGGCGACGCTGTTGGCAAAAGAACTGGATGCTG
2P5CS           CTGGTATATTCTGGGATAACGACAGTTTGGCGACGCTGTTGGCAAAAGAACTGGATGCTG
1P5CS           CTGGTATATTCTGGGATAACGACAGTTTGGCGACGCTGTTGGCAAAAGAACTGGATGCTG
4P5CS           CTGGTATATTCTGGGATAACGACAGTTTGGCGACGCTGTTGGCAAAAGAACTGGATGCTG
18P5CS          CTGGTATATTCTGGGATAACGACAGTTTGGCGACGCTGTTGGCAAAAGAACTGGATGCTG
21P5CS          TCGGCATATTCTGGGATAACGACAGTTTGGCGACGCTGTTGGCAAAAGAACTGGATGCTG
23P5CS          CTGGCATATTCTGGGATAACGACAGTTTGGCGACGCTGTTGGCAAAAGAACTGGATGCTG
16P5CS          CTGGCATATTCTGGGATAACGACAGTTTGGCGACGCTGTTGGCAAAAGAACTGGATGCTG
24P5CS          CTGGCATATTCTGGGATAACGACAGTTTGGCGACGCTGTTGGCAAAAGAACTGGATGCTG
22P5CS          CTGGTATATTCTGGGATAACGACAGTTTGGCGACGCTGTTGGCAAAAGAACTGGATGCTG
25P5CS          CTGGTATATTCTGGGATAACGACAGTTTGGCGACGCTGTTGGCAAAAGAACTGGATGCTG
14P5CS          CTGGTATATTCTGGGATAATGACAGTTTGGCGACGCTGTTGGCAAAAGAACTGGATGCTG
5P5CS           CTGGCATATTCTGGGATAACGACAGTTTGGCAACGCTGTTGGCGAAAGAACTGGATGCTG
27P5CS          CTGGTATATTCTGGGATAACGACAGTTTGGCAACGCTGTTGGCGAAAGAACTGGATGCTG
29P5CS          CTGGTATATTCTGGGATAACGACAGTTTGGCAACGCTGTTGGCGAAAGAACTGGATGCTG
3P5CS           CTGGTATATTCTGGGATAACGACAGTTTGGCAACGCTGTTGGCGAAAGAACTGGATGCTG
                  ** ************** *********** *********** ****************

26P5CS          ATCTACTTATCATGCTTAGTGATGTGGAGGGACTCTATAGTGGTCCACCGAGTGATCCTC
28P5CS          ATCTACTTATCATGCTTAGTGATGTGGAGGGACTCTATAGTGGTCCACCGAGTGATCCTC
19P5CS          ATCTACTTATCATGCTTAGTGATGTGGAGGGACTCTATAGTGGTCCACCGAGTGATCCTC
17P5CS          ATCTACTTATCATGCTTAGTGATGTGGAGGGACTCTATAGTGGTCCACCGAGTGATCCTC
12P5CS          ATCTACTTATCATGCTTAGTGATGTGGAGGGACTCTATAGTGGTCCACCGAGTGATCCTC
2P5CS           ATCTACTTATCATGCTTAGTGATGTGGAGGGACTCTATAGTGGTCCACCGAGTGATCCTC
1P5CS           ATCTACTTATCATGCTTAGTGATGTGGAGGGACTCTATAGTGGTCCACCGAGTGATCCTC
4P5CS           ATCTACTTATCATGCTTAGTGATGTGGAGGGACTCTATAGTGGTCCACCGAGTGATCCTC
18P5CS          ATCTACTTATCATGCTTAGTGATGTGGAGGGACTCTATAGTGGTCCACCGAGTGATCCTC
21P5CS          ATCTACTTATCATGCTTAGTGATGTGGAGGGACTCTATAGTGGTCCACCGAGTGATCCTC
23P5CS          ATCTACTTATCATGCTTAGTGATGTGGAGGGACTCTATAGTGGTCCACCGAGTGATCCTC
16P5CS          ATCTACTTATCATGCTTAGTGATGTGGAGGGACTCTATAGTGGTCCACCGAGTGATCCTC
24P5CS          ATCTACTTATCATGCTTAGTGATGTGGAGGGACTCTATAGTGGTCCACCGAGTGATCCTC
22P5CS          ATCTACTTATCATGCTTAGTGATGTGGAGGGACTCTATAGTGGTCCACCGAGTGATCCTC
25P5CS          ATCTACTTATCATGCTTAGTGATGTGGAGGGACTCTATAGTGGTCCACCGAGTGATCCTC
14P5CS          ATCTACTTATCATGCTTAGTGATGTGGAGGGACTCTATAGTGGTCCACCGAGTGATCCTC
5P5CS           ATCTTCTTATCATGCTTAGTGATGTGGAGGGACTCTATAGTGGTCCACCGAGCGATCCCC
27P5CS          ATCTTCTTATCATGCTTAGTGATGTGGAGGGACTCTATAGTGGTCCACCGAGCGATCCCC
29P5CS          ATCTTCTTATCATGCTTAGTGATGTGGAGGGACTCTATAGTGGTCCACCGAGCGATCCCC
3P5CS           ATCTTCTTATCATGCTTAGTGATGTGGAGGGACTCTATAGTGGTCCACCGAGCGATCCCC
                **** *********************************************** ***** *

26P5CS          AATCAAAGATTATCCACACCTACATCAATGAAAAACACGGGAAGCTAATTAATTTTGGGG
28P5CS          AATCAAAGATTATCCACACCTACATCAATGAAAAACACGGGAAGCTAATTAATTTTGGGG
19P5CS          AATCAAAGATTATCCACACCTACATCAATGAAAAACACGGGAAGCTAATTAATTTTGGGG
17P5CS          AATCAAAGATTATCCACACCTACATCAATGAAAAACACGGGAAGCTAATTAATTTTGGGG
12P5CS          AATCAAAGATTATCCACACCTACATCAATGAAAAACACGGGAAGCTAATTAATTTTGGGG
2P5CS           AATCAAAGATTATCCACACCTACATCAATGAAAAACACGGGAAGCTAATTAATTTTGGGG
1P5CS           AATCAAAGATTATCCACACCTACATCAATGAAAAACACGGGAAGCTAATTAATTTTGGGG
4P5CS           AATCAAAGATTATCCACACCTACATCAATGAAAAACACGGGAAGCTAATTAATTTTGGGG
18P5CS          AATCAAAGATTATCCACACCTACATCAATGAAAAACACGGGAAGCTAATTAATTTTGGGG
21P5CS          AATCAAAGATTATCCACACCTACATCAATGAAAAACACGGGAAGCTAATTAATTTTGGGG
23P5CS          AATCAAAGATTATCCACACCTACATCAATGAAAAACACGGGAAGCTAATTAATTTTGGGG
16P5CS          AATCAAAGATTATCCACACCTACATCAATGAAAAACACGGGAAGCTAATTAATTTTGGGG
24P5CS          AATCAAAGATTATCCACACCTACATCAATGAAAAACACGGGAAGCTAATTAATTTTGGGG
22P5CS          AATCAAAGATTATCCACACCTACATCAATGAAAAACACGGGAAGCTAATTAATTTTGGGG
25P5CS          AATCAAAGATTATCCACACCTACATCAATGAAAAACACGGGAAGCTAATTAATTTTGGGG
14P5CS          AATCAAAGATTATCCACACCTACATCAATGAAAAACACGGGAAGCTAATTAATTTTGGGG
5P5CS           AATCAAAGATTATCCACACCTACATTAATGAAAAACACGGGAAGCTAATTAATTTTGGGG
27P5CS          AATCAAAGATTATCCACACCTACATTAATGAAAAACACGGGAAGCTAATTAATTTTGGGG
29P5CS          AATCAAAGATTATCCACACCTACATTAATGAAAAACACGGGAAGCTAATTAATTTTGGGG
```

```
3P5CS          AATCAAAGATTATCCACACCTACATTAATGAAAAACACGGGAAGCTAATTAATTTTGGGG
               ************************* **********************************

26P5CS         AGAAGTCTCGTGTGGGAAGAGGTGGAATGCAAGCTAAGGTGGCAGCTGCTGTTACTGCTG
28P5CS         AGAAGTCTCGTGTGGGAAGAGGTGGAATGCAAGCTAAGGTGGCAGCTGCTGTTACTGCTG
19P5CS         AGAAGTCTCGTGTGGGAAGAGGTGGAATGCAAGCTAAGGTGGCAGCTGCTGTTACTGCTG
17P5CS         AGAAGTCTCGTGTGGGAAGAGGTGGAATGCAAGCTAAGGTGGCAGCTGCTGTTACTGCTG
12P5CS         AGAAGTCTCGTGTGGGAAGAGGTGGAATGCAAGCTAAGGTGGCAGCTGCTGTTACTGCTG
2P5CS          AGAAGTCTCGTGTGGGAAGAGGTGGAATGCAAGCTAAGGTGGCAGCTGCTGTTACTGCTG
1P5CS          AGAAGTCTCGTGTGGGAAGAGGTGGAATGCAAGCTAAGGTGGCAGCTGCTGTTACTGCTG
4P5CS          AGAAGTCTCGTGTGGGAAGAGGTGGAATGCAAGCTAAGGTGGCAGCTGCTGTTACTGCTG
18P5CS         AGAAGTCTCGTGTGGGAAGAGGTGGAATGCAAGCTAAGGTGGCAGCTGCTGTTACTGCTG
21P5CS         AGAAGTCTCGTGTGGGAAGAGGTGGAATGCAAGCTAAGGTGGCAGCTGCTGTTACTGCTG
23P5CS         AGAAGTCTCGTGTGGGAAGAGGTGGAATGCAAGCTAAGGTGGCAGCTGCTGTTACTGCTG
16P5CS         AGAAGTCTCGTGTGGGAAGAGGTGGAATGCAAGCTAAGGTGGCAGCTGCTGTTACTGCTG
24P5CS         AGAAGTCTCGTGTGGGAAGAGGTGGAATGCAAGCTAAGGTGGCAGCTGCTGTTACTGCTG
22P5CS         AGAAGTCTCGTGTGGGAAGAGGTGGAATGCAAGCTAAGGTGGCAGCTGCTGTTACTGCTG
25P5CS         AGAAGTCTCGTGTGGGAAGAGGTGGAATGCAAGCTAAGGTGGCAGCTGCTGTTACTGCTG
14P5CS         AGAAGTCTCGTGTGGGAAGAGGTGGAATGCAAGCTAAGGTGGCAGCTGCTGTTACTGCTG
5P5CS          AGAAGTCTCGTGTGGGAAGAGGTGGAATGCAAGCTAAGGTGGCAGCTGCTGTTACTGCTG
27P5CS         AGAAGTCTCGTGTGGGAAGAGGTGGAATGCAAGCTAAGGTGGCAGCTGCTGTTACTGCTG
29P5CS         AGAAGTCTCGTGTGGGAAGAGGTGGAATGCAAGCTAAGGTGGCAGCTGCTGTTACTGCTG
3P5CS          AGAAGTCTCGTGTGGGAAGAGGTGGAATGCAAGCTAAGGTGGCAGCTGCTGTTACTGCTG
               ************************************************************

26P5CS         CATCAAAGGGCGTACCTGCTGTAATTGCAAGTGGATTTGTAACAGATAGCATTATTAAGA
28P5CS         CATCAAAGGGCGTACCTGCTGTAATTGCAAGTGGATTTGTAACAGATAGCATTATTAAGA
19P5CS         CATCAAAGGGCGTACCTGCTGTAATTGCAAGTGGATTTGTAACAGATAGCATTATTAAGA
17P5CS         CATCAAAGGGCGTACCTGCTGTAATTGCAAGTGGATTTGTAACAGATAGCATTATTAAGA
12P5CS         CATCAAAGGGCGTACCTGCTGTAATTGCAAGTGGATTTGTAACAGATAGCATTATTAAGA
2P5CS          CATCAAAGGGCGTACCTGCTGTAATTGCAAGTGGATTTGTAACAGATAGCATTATTAAGA
1P5CS          CATCAAAGGGCGTACCTGCTGTAATTGCAAGTGGATTTGTAACAGATAGCATTATTAAGA
4P5CS          CATCAAAGGGCGTACCTGCTGTAATTGCAAGTGGATTTGTAACAGATAGCATTATTAAGA
18P5CS         CATCAAAGGGCGTACCTGCTGTAATTGCAAGTGGATTTGTAACAGATAGCATTATTAAGA
21P5CS         CATCAAAGGGCGTACCTGCTGTAATTGCAAGTGGATTTGTAACAGATAGCATTATTAAGA
23P5CS         CATCAAAGGGCGTACCTGCTGTAATTGCAAGTGGATTTGTAACAGATAGCATTATTAAGA
16P5CS         CATCAAAGGGCGTACCTGCTGTAATTGCAAGTGGATTTGTAACAGATAGCATTATTAAGA
24P5CS         CATCAAAGGGCGTACCTGCTGTAATTGCAAGTGGATTTGTAACAGATAGCATTATTAAGA
22P5CS         CATCAAAGGGCGTACCTGCTGTAATTGCAAGTGGATTTGTAACAGATAGCATTATTAAGA
25P5CS         CATCAAAGGGCGTACCTGCTGTAATTGCAAGTGGATTTGTAACAGATAGCATTATTAAGA
14P5CS         CATCAAAGGGCGTACCTGCTGTAATTGCAAGTGGATTTGTAACAGATAGCATTATTAAGA
5P5CS          CATCAAAGGGCGTACCTGCTGTAATTGCAAGTGGATTTGTAACAGATAGCATTATTAAGA
27P5CS         CATCAAAGGGCGTACCTGCTGTAATTGCAAGTGGATTTGTAACAGATAGCATTATTAAGA
29P5CS         CATCAAAGGGCGTACCTGCTGTAATTGCAAGTGGATTTGTAACAGATAGCATTATTAAGA
3P5CS          CATCAAAGGGCGTACCTGCTGTAATTGCAAGTGGATTTGTAACAGATAGCATTATTAAGA
               ************************************************************

26P5CS         TTATGCGAGGAGAGAAAATTGGTACACTTTTCCATAATGAAGCAAATGTCTGGGATTGTT
28P5CS         TTATGCGAGGAGAGAAAATTGGTACACTTTTCCATAATGAAGCAAATGTCTGGGATTGTT
19P5CS         TTATGCGAGGAGAGAAAATTGGTACACTTTTCCATAATGAAGCAAATGTCTGGGATTGTT
17P5CS         TTATGCGAGGAGAGAAAATTGGTACACTTTTCCATAATGAAGCAAATGTCTGGGATTGTT
12P5CS         TTATGCGAGGAGAGAAAATTGGTACACTTTTCCATAATGAAGCAAATGTCTGGGATTGTT
2P5CS          TTATGCGAGGAGAGAAAATTGGTACACTTTTCCATAATGAAGCAAATGTCTGGGATTGTT
1P5CS          TTATGCGAGGAGAGAAAATTGGTACACTTTTCCATAATGAAGCAAATGTCTGGGATTGTT
4P5CS          TTATGCGAGGAGAGAAAATTGGTACACTTTTCCATAATGAAGCAAATGTCTGGGATTGTT
18P5CS         TTATGCGAGGAGAGAAAATTGGTACACTTTTCCATAATGAAGCAAATGTCTGGGATTGTT
21P5CS         TTATGCGAGGAGAGAAAATTGGTACACTTTTCCATAATGAAGCAAATGTCTGGGATTGTT
23P5CS         TTATGCGAGGAGAGAAAATTGGTACACTTTTCCATAATGAAGCAAATGTCTGGGATTGTT
16P5CS         TTATGCGAGGAGAGAAAATTGGTACACTTTTCCATAATGAAGCAAATGTCTGGGATTGTT
24P5CS         TTATGCGAGGAGAGAAAATTGGTACACTTTTCCATAATGAAGCAAATGTCTGGGATTGTT
22P5CS         TTATGCGAGGAGAGAAAATTGGTACACTTTTCCATAATGAAGCAAATGTCTGGGATTGTT
25P5CS         TTATGCGAGGAGAGAAAATTGGTACACTTTTCCATAATGAAGCAAATGTCTGGGATTGTT
14P5CS         TTATGCGAGGAGAGAAAATTGGTACACTTTTCCATAATGAAGCAAATGTCTGGGATTGTT
```

5P5CS TTATGCGAGGAGAGAAAATTGGTACACTTTTCCATAATGAAGCAAATGTCTGGGATTGTT
27P5CS TTATGCGAGGAGAGAAAATTGGTACACTTTTCCATAATGAAGCAAATGTCTGGGATTGTT
29P5CS TTATGCGAGGAGAGAAAATTGGTACACTTTTCCATAATGAAGCAAATGTCTGGGATTGTT
3P5CS TTATGCGAGGAGAGAAAATTGGTACACTTTTCCATAATGAAGCAAATGTCTGGGATTGTT
****

26P5CS CGAAGGAGGTGACAACCCGTGAGATGGCAGTTGCTGCAAAAGATTGCTCCAGACATCTAC
28P5CS CGAAGGAGGTGACAACCCGTGAGATGGCAGTTGCTGCAAAAGATTGCTCCAGACATCTAC
19P5CS CGAAGGAGGTGACAACCCGTGAGATGGCAGTTGCTGCAAAAGATTGCTCCAGACATCTAC
17P5CS CGAAGGAGGTGACAACCCGTGAGATGGCAGTTGCTGCAAAAGATTGCTCCAGACATCTAC
12P5CS CGAAGGAGGTGACAACCCGTGAGATGGCAGTTGCTGCAAAAGATTGCTCCAGACATCTAC
2P5CS CGAAGGAGGTGACAACCCGTGAGATGGCAGTTGCTGCAAAAGATTGCTCCAGACATCTAC
1P5CS CGAAGGAGGTGACAACCCGTGAGATGGCAGTTGCTGCAAAAGATTGCTCCAGACATCTAC
4P5CS CGAAGGAGGTGACAACCCGTGAGATGGCAGTTGCTGCAAAAGATTGCTCCAGACATCTAC
18P5CS CGAAGGAGGTGACAACCCGTGAGATGGCAGTTGCTGCAAAAGATTGCTCAAGACATCTAC
21P5CS CGAAGGAGGTGACAACCCGTGAGATGGCAGTTGCTGCAAAAGATTGCTCCAGACATCTAC
23P5CS CGAAGGAGGTGACAACCCGTGAGATGGCAGTTGCTGCAAAAGATTGCTCCAGACATCTAC
16P5CS CGAAGGAGGTGACAACCCGTGAGATGGCAGTTGCTGCAAAAGATTGCTCCAGACATCTAC
24P5CS CGAAGGAGGTGACAACCCGTGAGATGGCAGTTGCTGCAAAAGATTGCTCCAGGCATCTAC
22P5CS CGAAGGAGGTGACAACCCGTGAGATGGCAGTTGCTGCAAAAGATTGCTCCAGGCATCTAC
25P5CS CGAAGGAGGTGACAACCCGTGAGATGGCAGTTGCTGCAAAAGATTGCTCCAGGCATCTAC
14P5CS C**A**AAGGAGGTGACAACCCGTGAGATGGCAGTTGCTGCAAAAGATTGCTCCAGACATCTAC
5P5CS C**A**AAGGAGGTGACAACCCGTGAGATGGCAGTTGCTGCAAAAGATTGCTC**A**AGACATCTAC
27P5CS C**A**AAGGAGGTGACAACCCGTGAGATGGCAGTTGCTGCAAAAGATTGCTC**A**AGACATCTAC
29P5CS C**A**AAGGAGGTGACAACCCGTGAGATGGCAGTTGCTGCAAAAGATTGCTCCAGACATCTAC
3P5CS C**A**AAGGAGGTGACAACCCGTGAGATGGCAGTTGCTGCAAAAGATTGCTCCAGGCATCTAC
* *** **********
