# Supplementary material for: Durum Wheat Roots Adapt to Salinity Remodeling the Cellular Content of Nitrogen Metabolites and Sucrose
Source: Front Plant Sci. 2017 Jan 9;7:2035. doi: 10.3389/fpls.2016.02035 (PMC5220018; doi:10.3389/fpls.2016.02035)
Supplement: Supplementary file 6 [file Table1.DOCX]

**Supplemental table 1.** Primer sequences for semi-quantitative RT-PCR.

| **Primers** | **Sequence (5’-3’)** |
|---|---|
| *Asn1*-F | TGTTGCCGTCGATCCAGGAAAATG |
| *Asn1*-R | GGCAAGCAGGACAGGACACCATCAAC |
| *Asn2*-F | ACGAGGCTGGCTGTAGAGGAC |
| *Asn2*-R | AACCGTTCTTATTTGCCTTTAGTAG |
| *Asn3*-F | AGAGCATCCTAGTCGAAACTGG |
| *Asn3*-R | ACGGACAAGTTGCAACCAAG |
| *P5CS*-F | CTGGYATATTCTGGGATAAY |
| *P5CS*-R | CTGTAGATGYCTKGARCAAT |
| *Actin*-F | ACTACTGGTATTGTGCTCGAC |
| *Actin*-R | TCTTACAATTTCCCGCTCTG |
